# The *NF2* tumor suppressor merlin interacts with Ras and RasGAP, which may modulate Ras signaling

**DOI:** 10.1038/s41388-019-0883-6

**Published:** 2019-07-16

**Authors:** Yan Cui, Susann Groth, Scott Troutman, Annemarie Carlstedt, Tobias Sperka, Lars Björn Riecken, Joseph L. Kissil, Hongchuan Jin, Helen Morrison

**Affiliations:** 1 Leibniz Institute on Aging, Fritz Lipmann Institute, Beutenbergstr. 11, 07745 Jena, Germany; 2 Department of Molecular Medicine, the Scripps Research Institute, 130 Scripps Way, Jupiter, FL 33458 USA; 3 Laboratory of Cancer Biology, Key Laboratory of Biotherapy, Sir Run Run Shaw Hospital, Medical School of Zhejiang University, Hangzhou, 310016 China

**Keywords:** Growth factor signalling, Cancer genetics, Biochemistry, Cancer

## Abstract

Inactivation of the tumor suppressor NF2/merlin underlies neurofibromatosis type 2 (NF2) and some sporadic tumors. Previous studies have established that merlin mediates contact inhibition of proliferation; however, the exact mechanisms remain obscure and multiple pathways have been implicated. We have previously reported that merlin inhibits Ras and Rac activity during contact inhibition, but how merlin regulates Ras activity has remained elusive. Here we demonstrate that merlin can directly interact with both Ras and p120RasGAP (also named RasGAP). While merlin does not increase the catalytic activity of RasGAP, the interactions with Ras and RasGAP may fine-tune Ras signaling. In vivo, loss of RasGAP in Schwann cells, unlike the loss of merlin, failed to promote tumorigenic growth in an orthotopic model. Therefore, modulation of Ras signaling through RasGAP likely contributes to, but is not sufficient to account for, merlin’s tumor suppressor activity. Our study provides new insight into the mechanisms of merlin-dependent Ras regulation and may have additional implications for merlin-dependent regulation of other small GTPases.

## Introduction

Neurofibromatosis type 2 (NF2) is a multiple tumor syndrome that results from loss-of-function mutations in the *NF2* gene [1]. The hallmark of NF2 is the development of bilateral vestibular schwannomas, although NF2 patients can develop other schwannomas, meningiomas, ependymomas, and astrocytomas [1]. Biallelic *NF2* inactivation also occurs in various sporadic tumors, especially in schwannomas, meningiomas, malignant mesothelioma, and ependymomas [2].

The *NF2* gene encodes a 70-kDa protein called merlin that belongs to the ezrin, radixin, and moesin (ERM) protein family. Merlin and ERM proteins share a similar domain organization, containing an N-terminal FERM domain, a central α-helical domain and a C-terminal tail [2]. However, merlin and ERM proteins appear to act antagonistically on cell proliferation and transformation [3]. Merlin is long recognized as a critical mediator of contact inhibition of proliferation; its exact mechanisms remain unclear, although multiple pathways and mechanisms have been implicated, including regulation of Ras pathway activity [2].

Sustained activity of the Ras pathways, caused by mutations in Ras or/and its pathway components, promotes cellular transformation and drives tumorigenesis [4]. Loss of merlin functions likewise results in cellular transformation and is associated with sustained activation of the Ras pathways [5, 6]. Conversely, overexpression of merlin can counteract Ras-induced transformation [7, 8], supporting the relevance of merlin-mediated regulation of the Ras pathways, which may occur at multiple levels (see later in “Discussion”). Whilst we have previously shown that merlin interferes with the activation of the small GTPases Ras and Rac [5], the exact mechanism by which merlin interferes with Ras activation remains largely unknown.

Ras cycles between an inactive GDP-bound and an active GTP-bound state [4]. However, the spontaneous GTP-loading and the intrinsic Ras-GTP hydrolysis are very slow. Rather, the activity of Ras is directly controlled by guanine nucleotide exchange factors (GEFs) that catalyze the exchange of GDP for GTP, and by GTPase activating proteins (GAPs) that stimulate the GTPase activity [4]. Several Ras GAPs exist in mammals, among which p120RasGAP (also named RasGAP, encoded by *RASA1*) and neurofibromin (encoded by *NF1*) are prototypic, coexisting in most cell types [9]. In this study, we demonstrate that merlin can bind both Ras and RasGAP. Whereas merlin does not appear to directly regulate the catalytic activity of RasGAP, the interactions with Ras and RasGAP may fine-tune Ras signaling in space and time.

## Results

### *Nf2*^−/−^ mouse Schwann cells exhibit elevated Ras activity and require Ras activity for focus formation

**Fig. 1:**
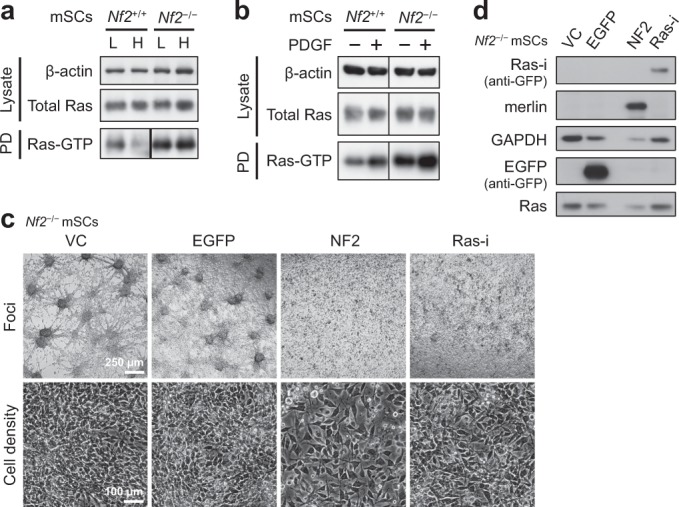
*Nf2*^−/−^ mSCs exhibit elevated Ras activity and depend on Ras activity for focus formation. **a**, **b** Ras activity in *Nf2*^+/+^ and *Nf2*^−/−^ mSCs was analyzed by GST-Raf1 RBD pulldown followed by western blotting. Spliced lanes are from the same exposure of the same blot. **a** Cells were cultured at low (L) or high (H) cell density before lysis. **b** Cells were growth factors starved overnight and lysed following stimulation with 10 ng/ml PDGF-BB for 2 min, or without stimulation. **c**, **d** *Nf2*^−/−^ mSCs were transduced with lentiviral vectors encoding the indicated transgenes and selected with blasticidin to eliminate nontransduced cells. Cell density (monolayer) and foci were photographed 5 and 6 days after transduction, respectively (**c**); transgene expression was verified by western blotting (**d**). PD: pulldown; VC: vector control. See also Fig. S1. Representative data from one out of two independent experiments are shown for all panels

We used immortalized *Nf2*^−/−^ and the matched *Nf2*^+/+^ mouse Schwann cells [10] (mSCs; Fig. S1a) to analyze Ras activity. *Nf2*^−/−^ mSCs showed elevated Ras-GTP levels, regardless of cell density (Fig. 1a). This too was the case when the cells were under growth factor-starved condition or further stimulated with PDGF-BB (Fig. 1b), a growth factor contributing to Schwann cell survival and proliferation [11–13]. Loss of contact inhibition in *Nf2*^−/−^ mSCs was evidenced by multiple foci formation in culture (see Fig. 1c). To investigate the contribution of Ras hyperactivity to *Nf2* loss-induced focus formation, we constructed a Ras-inhibitory protein (Ras-i) consisting of EGFP, the Ras-binding domain (RBD) and the cysteine-rich domain (CRD) of Raf1, and the GAP-related domain (GRD) of NF1 (Fig. S1b). The RBD-CRD of Raf1 has been shown to be able to block endogenous Ras-GTP signaling [14], whereas NF1 GRD can potently promote Ras-GTP inactivation. The combination of both, we reasoned, should be capable of counteracting Ras hyperactivity. Indeed, introduction of Ras-i into *Nf2*^−/−^ mSCs lowered Ras-GTP level as expected (Fig. S1c), reduced focus formation, and improved contact inhibition, although not as efficiently as the reconstitution of merlin (Fig. 1c). Of note, Ras-i was poorly expressed (Fig. 1d) and the expression was rapidly lost when the cells were further expanded (not shown). These data suggest that Ras activity is required for focus formation in *Nf2*^−/−^ mSCs and suppression of Ras activity is likely an important part of merlin’s tumor suppressor functions.

### The FERM domain of merlin can directly interact with Ras

**Fig. 2:**
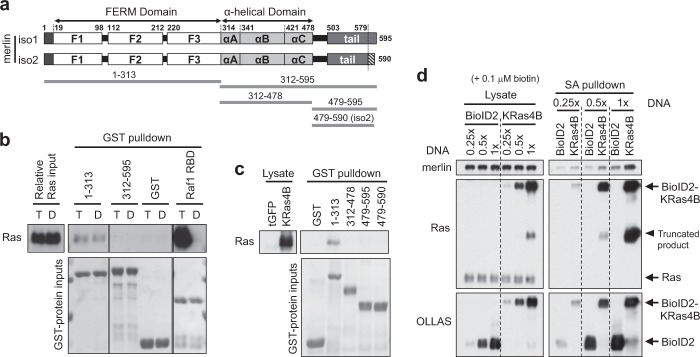
Merlin can directly interact with Ras. **a** Domain organization of merlin isoform 1 and 2 (iso1/2) and GST-fusion fragments used for pulldown. Note that aa 1–579 are identical in iso1 and 2. **b** Western blot analysis of GST-merlin fragments pulldown of HRas 1–166 preloaded with GDP (D) or GTPγS (T). GST-Raf1 RBD was included for comparison. All proteins were purified from *E. coli*. GST-fusion protein inputs were stained with Ponceau S. **c** Western blot analysis of GST-merlin fragments pulldown of KRas4B overexpressed in 293 cells by transient transfection. TurboGFP (tGFP) transfected sample shows endogenous total Ras, much lower than KRas4B. Representative data from one out of two independent experiments are shown**. d** Varying amounts of constructs for proximity biotinylation (with an N-terminal OLLAS tag) were transfected into 293 cells; ~24 h later, media was replenished with 0.1 µM biotin; ~48 h after transfection, cells were lysed for streptavidin (SA) pulldown, analyzed by western blotting. Note that Ras and OLLAS were sequentially probed without stripping, taking advantage of different host species for the antibodies. Prior signals were inactivated by H_2_O_2_ treatment before the second round of probing. See also Fig. S3

Merlin and ERM proteins are regarded as physiological counter players [5, 15, 16]. We have previously shown that ezrin is actively involved in Ras activation by directly binding to both Ras and Son of Sevenless (SOS), a major Ras GEF [15, 16]. The F1 subdomain of FERM is an ubiquitin/Raf1-RBD-like domain [17] and the F1–F2 subdomains of ezrin FERM appear to mediate Ras binding [15, 18], suggesting that merlin may do likewise. Indeed, merlin FERM (amino acids [aa] 1–313), but not the α-helical-tail domains (aa 312–595), could pull down purified HRas 1–166 (Fig. 2a, b). In contrast to Ras effectors like Raf proteins [4], merlin did not show any preference for either GTP- or GDP-loaded Ras, and the binding was apparently very weak. Further truncation revealed that a part of merlin’s F1 subdomain (aa 1–73) was sufficient to bind Ras (Fig. S2). To test whether merlin can bind other Ras isoforms, we performed pulldown with nontagged full-length (FL) KRas4B overexpressed in 293 cells, the most abundant and essential Ras isoform [19, 20]. The results illustrated that merlin FERM can also bind KRas4B and that the isolated α-helical domain or the tail from merlin isoform 1 or 2 (iso1/2, see later) cannot (Fig. 2c). An attempt was made to test merlin’s binding to FL HRas and NRas under the same condition; however, both bound to glutathione sepharose nonspecifically (not shown). Nevertheless, because HRas 1–166 represents the highly conserved G-domain, almost identical in H-, K-, and NRas [20], the results suggest that merlin is likely able to bind all Ras isoforms directly.

### Merlin interacts with Ras in vivo

Given the weak interaction between merlin and Ras, it is difficult to demonstrate the in vivo interaction by co-immunoprecipitation (Co-IP). We therefore employed a proximity biotinylation approach, mediated by the promiscuous biotin ligase BioID2 [21]. Expression of BioID2-KRas4B versus BioID2 alone in 293 cells resulted in clearly increased biotinylation of endogenous merlin (Fig. S3a). Even when biotin concentration was reduced to 0.1 μM (a very low concentration for this type of assay) and BioID2-KRas4B was expressed much lower than endogenous Ras by reducing transfected DNA amounts, increased biotinylation of merlin was still detectable (Figs. 2d and S3b)—suggesting that KRas4B and merlin are at close proximity in vivo. Together with the in vitro binding data, the in vivo interaction between Ras and merlin could be inferred.

### Merlin interacts with RasGAP

**Fig. 3:**
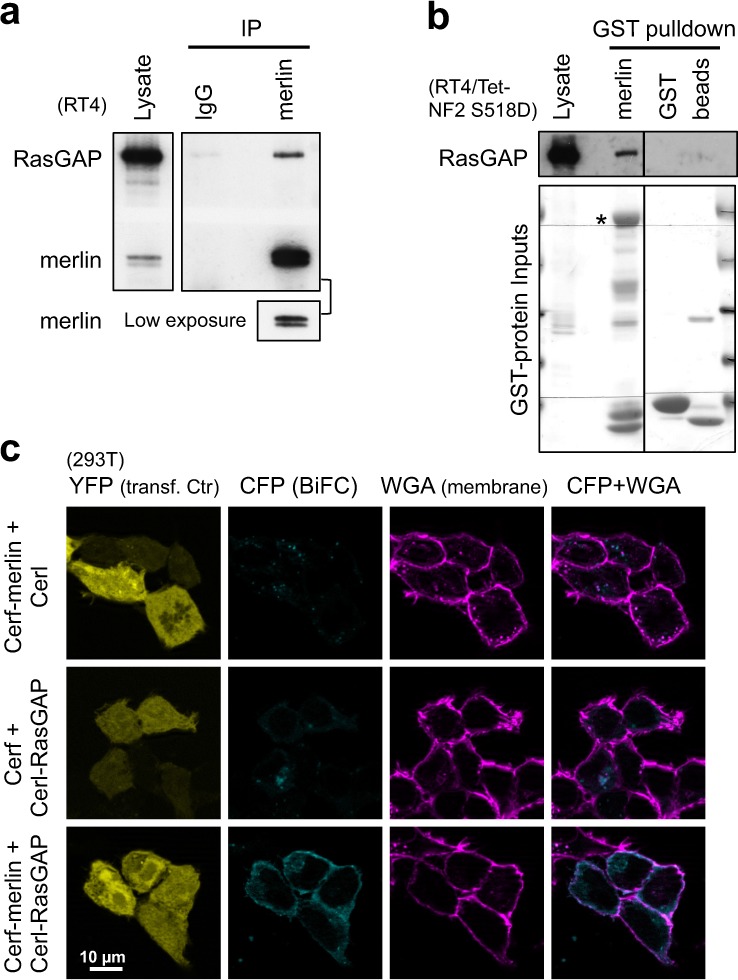
Merlin interacts with RasGAP. **a** Western blot analysis of Co-IP of RasGAP with merlin. **b** Western blot analysis of GST-merlin pulldown of RasGAP. Note that GST-merlin S518A and lysate from RT4/Tet-NF2 S518D cells were used here. Spliced lanes are from the same exposure of the same blot. GST-fusion protein inputs were stained with Ponceau S. Asterisks indicate the correct band. **c** Bimolecular fluorescence complementation (BIFC) analysis of merlin and RasGAP interaction. Constructs fused with a split cerulean protein (Cerf or Cerl) were cotransfected with pEYFP-C1 (transfection control) into 293T cells. Approximately 48 h later, cells were fixed and stained with wheat germ agglutinin (WGA)-Texas Red-X to outline outside membranes. CFP/YFP: cyan/yellow fluorescent protein. Representative data from one out of at least two independent experiments are shown for all panels

It has been shown that the GAP activity (toward Ras) from cell extracts was proportional to cell density in culture, suggesting that Ras GAPs may be involved in contact inhibition [22]. We hypothesized that merlin may recruit a RasGAP to negatively regulate Ras, reminiscent of the Ras-activating complex formed by ezrin and SOS [15, 16]. Indeed, RasGAP could be co-immunoprecipitated with endogenous merlin from confluent RT4 cells (a rat schwannoma cell line; Fig. 3a), whereas neurofibromin was poorly extracted with the same lysis buffer and thus not detectable (not shown). Moreover, GST-merlin could efficiently pull down endogenous RasGAP from RT4 cell lysate (Fig. 3b), further supporting the interaction and leading us to focus on RasGAP in this work.

### Merlin interacts with RasGAP predominantly at the plasma membrane

To validate merlin:RasGAP interaction and localize it within a cellular context, we attempted to stain the endogenous proteins, but failed to obtain confident signals. The bimolecular fluorescence complementation (BiFC) approach was then employed, using two complimentary fragments (Cerf and Cerl) of a split cerulean fluorophore [23, 24]. Coexpression of Cerf-merlin and Cerl-RasGAP resulted in reconstituted cerulean fluorescence; outperforming the background fluorescence signals from the control combinations (Fig. 3c), supporting the specificity of merlin:RasGAP interaction. The fluorescence was predominant at the plasma membrane, consistent with the reported subcellular localization of active merlin [25] and where Ras activity regulation should occur.

### Both the FERM and the tail domains of merlin interact with RasGAP

**Fig. 4:**
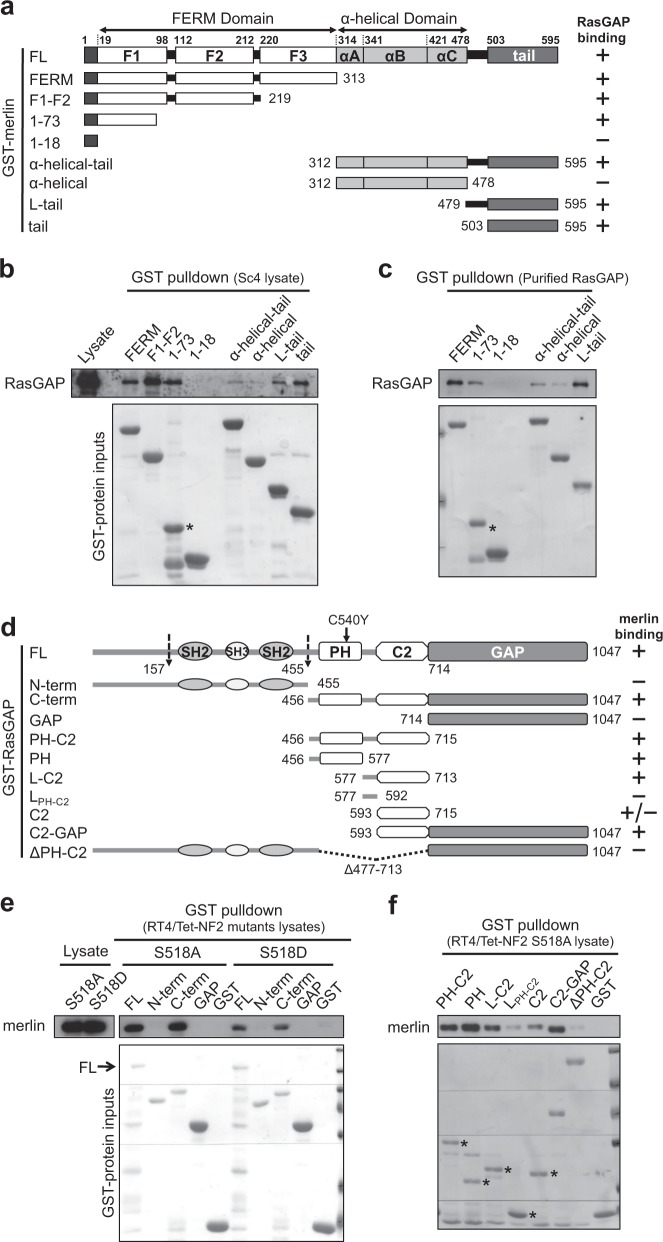
Merlin FERM and tail interact with the PH-C2 domains of RasGAP. **a** Domain organization of merlin and GST-fusion fragments used for pulldown. **b**, **c** Western blot analysis of GST-merlin fragments pulldown of RasGAP from Sc4 cell lysate (**b**) or full-length (FL) RasGAP purified from *E. coli* (**c**). **d** Domain organization of RasGAP and GST-fusion fragments used for pulldown. The locations of two caspase cleavage sites [57] and a patient-derived mutation C540Y are depicted. **e, f** Western blot analysis of GST-RasGAP fragments pulldown of merlin from indicated cell lysates. GST-fusion protein inputs were stained with Ponceau S. Asterisks indicate correct bands. Representative data from one out of at least two independent experiments are shown for **b** and **f**

Pulldown was performed using GST-merlin fragments, in order to define the domains mediating merlin:RasGAP interaction (Fig. 4a, b). Both the FERM domain and the α-helical-tail domains could pull down RasGAP independently, while the former was more efficient. Progressive truncation located two RasGAP-binding sites, one in the F1 subdomain and the other in the tail. The F3 subdomain, a PTB/PH-like domain [17], is unlikely involved in the binding, the deletion of which rather enhanced the binding. The first 18 amino acids (aa 1–18), unique to merlin and not present in ERMs, were unable to bind RasGAP on their own. For determining whether merlin can directly bind RasGAP, we used GST-tagged merlin fragments to pull down purified FL RasGAP in vitro (Fig. 4c), therein confirming that both the aa 1–73 fragment and the tail of merlin are able to bind RasGAP directly and independently.

### The PH-C2 domains of RasGAP interact with merlin

Reciprocal GST pulldown revealed that, FL RasGAP, as well as the C-terminal fragment (C-term), could efficiently pull down merlin from cell lysates, whereas the N-terminal fragment and the GAP domain could not (Fig. 4d, e)—suggesting that the merlin-binding site is within the PH-C2 region. Of note, RasGAP’s binding to the nonphosphorylatable merlin S518A mutant (active) was much stronger than to the phospho-mimicking S518D mutant (inactive), likely due to a conformational effect [26]. We further confirmed that the isolated PH-C2 fragment was indeed able to bind merlin (Fig. 4f). Moreover, both the PH and the C2 domains could independently bind merlin, suggesting multiple merlin contact sites within the PH-C2 region. Conversely, deletion of the PH-C2 domains from FL RasGAP essentially eliminated merlin binding.

Far-western blotting was performed to corroborate the pulldown results, by incubating purified merlin with GST-RasGAP fragments immobilized on a nitrocellulose membrane (Fig. S4a, b). Consistent with the GST pulldown data, all the PH domain-containing fragments showed strong interaction with merlin, whereas the GAP domain and the partial C2 domain (aa 606–648) did not exhibit any binding. Conversely, deletion of the PH domain greatly reduced merlin binding, whereas partial deletion of the C2 domain had no effect. Viewed together, these results support a direct binding between merlin and RasGAP, mediated primarily by the PH domain. Incidentally, C540Y, a capillary malformation-arteriovenous malformation patient-derived mutation within the PH domain [27], did not impair merlin binding, suggesting that C540 may not be directly involved in the binding. C540Y more likely impairs protein folding, as the protein yields were greatly reduced (see Ponceau staining of the 158–1047 fragments in Fig. S4b).

We further tested the importance of the PH domain for merlin binding in cotransfected Sc4 cells (a merlin-deficient line) and learned that merlin could be co-immunoprecipitated with either EGFP-RasGAP or EGFP-PH, but not with EGFP-RasGAP ΔPH (Fig. S4c); demonstrating that the PH domain determines the interaction with merlin in vivo.

### Merlin isoform 2 also binds RasGAP

Merlin has two major isoforms, with the only difference at the C-terminus [28] (Fig. 2a). An interaction between the FERM and the tail in merlin iso1 renders a relatively closed conformation [26], whereas merlin iso2 has a relatively open conformation due to lack of such interaction [29]. Of note, merlin’s α-helical domain may contribute more to the closed conformation [30, 31]; thus, the difference in the conformations between the isoforms is more likely localized. To compare their binding to RasGAP, we overexpressed their S518A mutants (to prevent a potential effect by phosphorylation) in 293 cells and performed GST pulldown—they bound similarly to RasGAP (Fig. S5a). As merlin’s binding to angiomotin and lipids induces conformation opening [26, 32], we also performed pulldown with merlin expressed in *E. coli* and different binding buffers (Fig. S5b). These results did reveal a more efficient binding to RasGAP by merlin iso2 under two buffer conditions, further supporting the conformational effect.

### RasGAP may prefer to bind merlin rather than ERMs

In order to test whether RasGAP also binds ERMs, GST pulldown was performed with the lysate from 293 cells, which express all ERMs and merlin (Fig. S6). While unable to detect any binding to ezrin or moesin, we detected a weak binding to radixin, relatively less efficient than to merlin. Thus, RasGAP appears to prefer to bind merlin over ERMs, in line with the less conserved α-helical-tail domains between merlin and ERMs [28]. Of note, ERMs have a more closed conformation than merlin iso1 [26], which can be opened by lipid binding and subsequent phosphorylation at T567/564/558, respectively [33]. Given that the conformation states are unknown, further experiments using open conformation constructs with an identical epitope tag will help to draw a clearer conclusion.

### NF2 patient-derived mutations impair merlin’s interaction with RasGAP in vivo

The nontruncating mutations identified in NF2 patients are mostly concentrated within the F1 subdomain and the tail of merlin [34]. Two pathogenic mutations in the F1 subdomain—L64P and ΔF96—that most likely impair merlin folding [35, 36] were chosen and tested as to whether they impair the interaction with Ras or RasGAP. For in vitro binding, we chose to purify merlin aa 1–340 (wild type and the mutants), because FL merlin is difficult to purify from *E. coli*. Both mutants exhibited enhanced binding to Ras (Fig. S7a), whereas they bound to RasGAP similarly to the WT (Fig. S7b), suggesting that these mutation sites may not be directly involved in the binding.

**Fig. 5:**
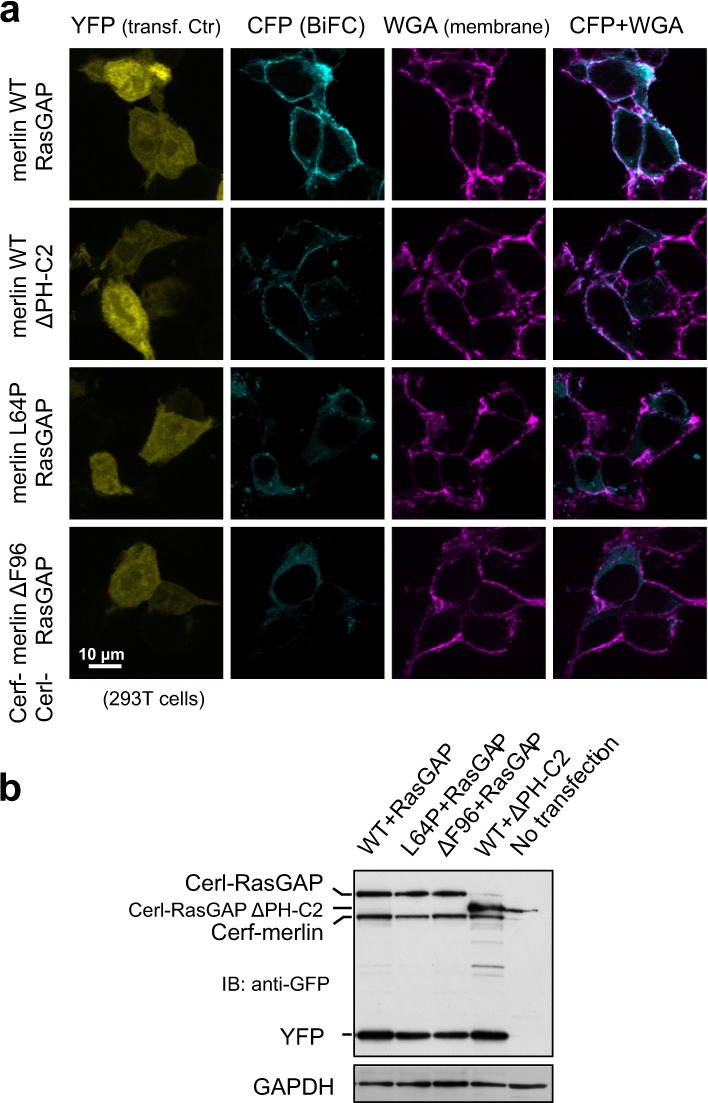
NF2 patient-derived mutations impair merlin:RasGAP interaction in vivo. **a** BiFC analysis of merlin and RasGAP interaction (WT and mutants). RasGAP ∆PH-C2 served as a binding-deficient control. See also legend for Fig. 3c. Note that the BiFC signals from merlin mutants were not only weak, but also relocated from the membrane to the cytoplasm. **b** Expression of BiFC constructs in a parallel transfection experiment validated by western blotting. The polyclonal GFP antibody detects both fragments of the split cerulean (Cerf/Cerl) and EYFP. Representative data from one out of three independent experiments are shown for **a** and **b**

Because the in vitro binding experiments may not reflect the in vivo situation in the context of FL merlin, BIFC experiments were performed to assess the mutations’ effect on merlin:RasGAP interaction (Fig. 5a). Both mutations caused markedly decreased BiFC efficiency, down to the negative control level (Cerf-merlin + Cerl-RasGAP ΔPH-C2). Although we cannot exclude an effect from different expression levels of the constructs (Fig. 5b), the BiFC signals from merlin mutants were predominantly displaced from the plasma membrane. Taken together, these mutations potentially impair a productive merlin:RasGAP interaction in different ways. First, the mutations may cause decreased expression and stability of merlin. Second, merlin mutants might form an ineffective interaction with RasGAP, away from the normal plasma membrane sites of activity.

### RasGAP is constitutively active and merlin does not increase the GAP activity in vitro

**Fig. 6:**
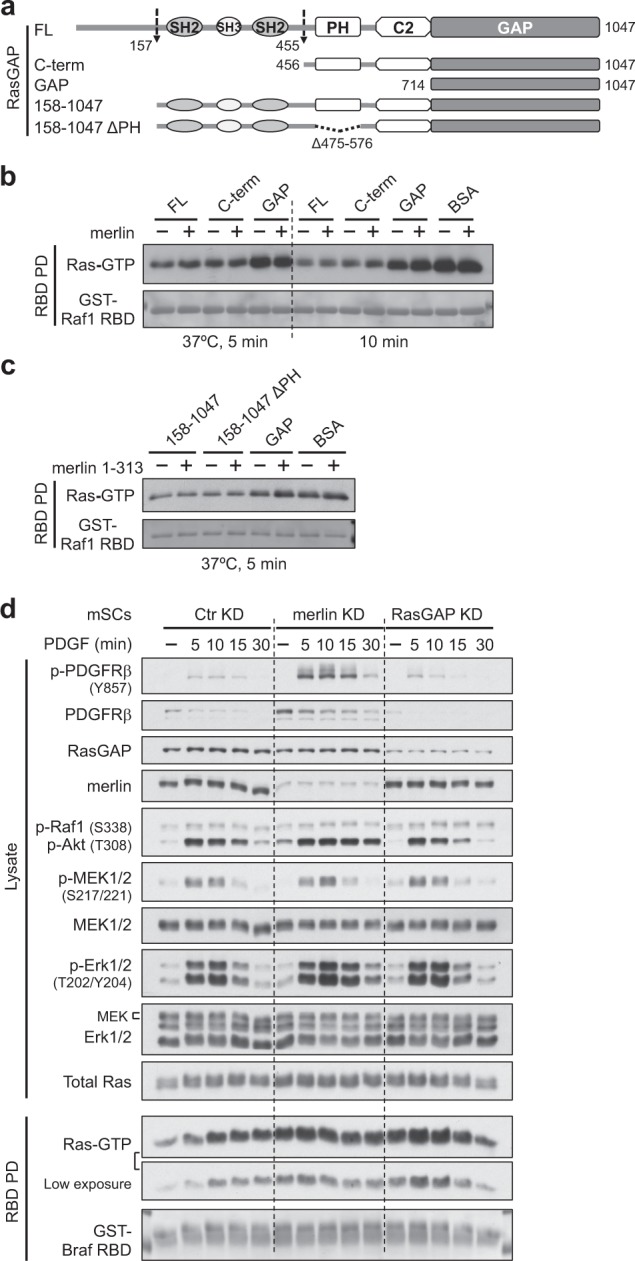
Merlin cannot increase RasGAP activity in vitro, but suppresses PDGF-BB activated Ras signaling in vivo. **a** RasGAP fragments used for in vitro GAP activity experiments. **b**, **c** Equal molar amounts of RasGAP fragments were preincubated with or without equal molar amounts of merlin (FL in **b**, aa 1–313 in **c**) for 1 h at room temperature and then used to catalyze the hydrolysis of GTP-loaded HRas 1–166. BSA was used as a noncatalyzing control. Remaining Ras-GTP was detected by GST-Raf1 RBD pulldown followed by western blotting. All proteins were purified from *E. coli*. See also Fig. S8a, b. **d** Cells were seeded and cultured for ~20 h (close to confluence), then growth factor starved overnight, then stimulated with 10 ng/ml PDGF-BB and lysed at indicated time points for Ras-GTP pulldown by GST-Braf RBD, analyzed by western blotting. Note that p-MEK, p-Erk, MEK, and Erk were sequentially probed without stripping, taking advantage of different host species for phosphorylation-specific and pan antibodies. Phosphorylation-specific signals were inactivated by H_2_O_2_ treatment before probing MEK. Note that uneven Erk signals were due to steric interference from previously bound antibodies (p-Erk signals were very strong before H_2_O_2_ treatment). Representative data from one out of two (**b**, **c**) or three (**d**) independent experiments are shown

The PH domain of RasGAP has been reported to bind the GAP domain and inhibit its activity [37]. Merlin can directly bind to Ras and the PH-C2 domains of RasGAP, raising the possibility that merlin may increase RasGAP activity by releasing RasGAP from autoinhibition and/or facilitating Ras and the GAP domain interaction. Therefore the in vitro GAP activity was tested using purified merlin, Ras (preloaded with GTP), and RasGAP (FL and fragments; Figs. 6a, b and S8a). The amount of remaining Ras-GTP after GAP-stimulated hydrolysis, which negatively correlates with GAP activity, was determined by a GST-Raf1 RBD pulldown-based approach. Yet, the presence of merlin did not increase, but rather slightly interfered with GAP activity. Nevertheless, we noticed that RasGAP FL and the C-term were much more active than the isolated GAP domain, indicating that the PH-C2 part contributes to, rather than inhibits, GAP activity—agreeing with an earlier study showing that RasGAP FL is around ten times more active than the GAP domain alone [38].

In testing whether the PH domain is autoinhibitory, we purified RasGAP aa 158–1047 (for better yield than the FL) and the PH-deleted counterpart, and tested the in vitro GAP activity in the absence and presence of merlin FERM (in case merlin FL may not interact with RasGAP efficiently in vitro, because of a relatively closed conformation; Figs. 6a, c and S8b). As seen with merlin FL, merlin FERM likewise failed to increase the GAP activity of any included RasGAP fragment. Deletion of the PH domain essentially had no effect on the GAP activity, while the isolated GAP domain was less active than the C2-GAP-containing fragments. We therefore conclude that RasGAP is constitutively active, the PH domain does not confer autoinhibition, the C2 domain is required for full GAP activity, and merlin cannot increase the GAP activity in vitro.

### Loss of merlin leads to more sustained activation of the downstream effectors in PDGF-BB signaling than loss of RasGAP

Although merlin appears unable to increase RasGAP activity in vitro, our previous data suggest that it can inhibit Ras activity in vivo (see Fig. 1a, b). Meanwhile, RasGAP is a well-known negative regulator of Ras. Indeed, there was elevated and prolonged Ras activation in confluent RasGAP-knockout (*Rasa1*^−/−^) mouse embryonic fibroblasts (MEFs) [39] following PDGF-BB stimulation (Fig. S8c). The phenotypes of merlin or RasGAP deficiency regarding Ras-GTP inactivation and tumorigenicity were further compared by generating *Nf2*^+/+^ mSCs derivatives with stable knockdown (KD) of merlin or RasGAP. We first analyzed PDGF-BB-induced Ras signaling in the cultures close to confluence (Fig. 6d). While the Ras-GTP dynamics varied between individual experiments, overall increased Ras-GTP levels in merlin-KD and RasGAP-KD mSCs were consistently observed. This too was the case for elevated expression of PDGFRβ and prolonged activation of Akt, MEK1/2, and Erk1/2 in merlin-KD mSCs—two prominent Ras effector pathways driving tumorigenesis [4]; Ras protein level also appeared to be elevated in merlin-KD mSCs, when normalized to the total protein level (Fig. S8d). By contrast, PDGFRβ signaling was terminated more rapidly in RasGAP-KD mSCs; although MEK1/2 and Erk1/2 were activated to a higher extent, they were deactivated similarly to the control cells. Thus, both merlin and RasGAP appear to suppress PDGF-BB-induced Ras signaling; however, loss of merlin leads to more sustained activation of the downstream effectors.

### Loss of RasGAP is not sufficient to promote tumorigenic growth in an orthotopic model

RasGAP has been previously implicated in contact inhibition [40] and we observed that *Rasa1*^−/−^ MEFs formed multiple foci after 2–3 weeks of culture (Fig. S9a), suggesting that RasGAP might be a relevant effector of merlin for contact inhibition.

**Fig. 7:**
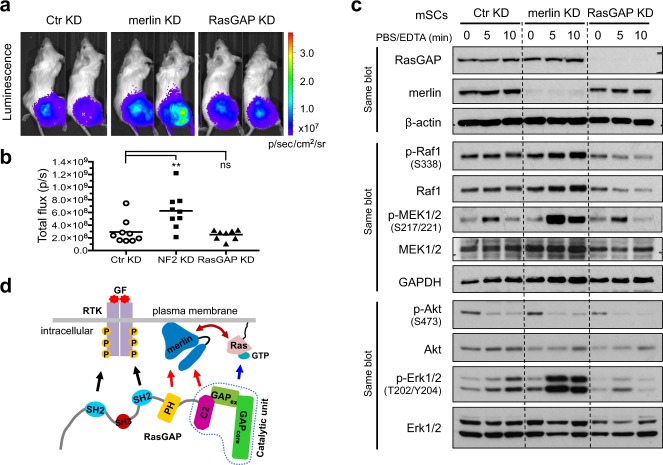
Comparison of RasGAP loss with merlin loss in tumorigenicity and detachment-induced signaling. **a**, **b** Bioluminescence imaging of orthotopic tumors formed by mSCs/Luc derivatives. Cells were injected into sciatic nerves of NOD/SCID mice (at one side) and tumor growth was monitored by measuring luciferase signals weekly for a total of 3 weeks. Endpoint data (images of representative animals, luciferase total flux from each animal, and the mean) are shown. **P ≤ 0.01; ns, nonsignificant; ordinary one-way ANOVA followed by Tukey’s multiple comparisons test. See also Fig. S9c. **c** Western blot analysis of signaling profiles of mSC derivatives under a detaching condition. Cells were treated with PBS/5 mM EDTA, harvested at indicated time points. Stripping was performed between sequential probing of phosphorylated and total protein level of the same target. Representative data from one out of two independent experiments are shown. **d** The model wherein the presence of merlin modulates Ras signaling. The GAP domain consists of two parts: GAP_extra_ and GAP_core_ [58]; the C2-GAP domains constitute the catalytic unit promoting Ras-GTP hydrolysis into Ras-GDP. The SH2-SH3-SH2 domains mediate RasGAP translocation from the cytoplasma to the plasma membrane through binding the consensus phosphotyrosine in various activated receptor tyrosine kinases (RTKs). Merlin FERM and tail interact with the PH-C2 domains of RasGAP, whereas the FERM also interacts with Ras (GTP-independent). Merlin may recruit RasGAP in concert with activated RTKs, or independently, to fine-tune Ras-GTP inactivation. Note that merlin might not simultaneously bind to both Ras and RasGAP. GF: growth factor

Next, orthotopic transplantations were conducted to assess the in vivo tumorigenic potential of merlin-KD and RasGAP-KD mSCs. We first generated *Nf2*^+/+^ mSCs/Luc that stably express a luciferase to enable bioluminescence imaging, and established the derivatives with stable KD of merlin or RasGAP (Fig. S9b). The cells were transplanted into sciatic nerves of nude mice and tumor growth monitored weekly for a total of 3 weeks. While KD of merlin promoted tumor growth, KD of RasGAP did not (Figs. 7a, b and S9c).

### Loss of merlin leads to more profound activation of MEK1/2–Erk1/2 in detachment-induced signaling than loss of RasGAP

Because anchorage-independent growth correlates with tumorigenicity [41, 42], it was prudent to analyze the signaling profiles of merlin-KD and RasGAP-KD cells under a detaching condition. Upon detachment, we expect that the Ras effector pathways to be uncoupled from Ras—this phenomenon is supposed to ensure that cells are unable to survive or proliferate in erroneous sites, a control mechanism lost in transformed cells [43]. For detachment, the cells were treated with PBS/5 mM EDTA, resulting in transient activation of MEK1/2 and more sustained activation of Erk1/2 (Fig. 7c), a response likely contributing to transient protection of the cells from anoikis [44]. Intriguingly, merlin-KD cells showed markedly increased MEK1/2–Erk1/2 activity, while the response of RasGAP-KD cells was comparable to that of control-KD cells. By contrast, detachment caused a rapid decrease of Akt activity in all tested cells. These results indicate that in addition to the merlin:RasGAP interaction upstream of Ras, merlin also targets the Ras effector pathways, particularly the MEK1/2–Erk1/2 branch—in the absence of merlin, the normal step to attenuate this pathway is lost, leading to an overactivation of MEK1/2 and Erk1/2.

## Discussion

Herein, we demonstrate that merlin directly interacts with both Ras and RasGAP. Merlin binds to RasGAP’s PH-C2 domains, whereas the PH and the C2 domains have been previously implicated in contact inhibition: only reconstitution of *Rasa*^−*/*−^ MEFs with RasGAP FL—but not with either PH or partial C2 domain-deleted—restored contact inhibition [40]. As PH domain deletion did not affect RasGAP’s catalytic activity in vitro, this rather indicates that loss of binding, e.g., toward merlin, may underlie the inability of PH-deleted RasGAP to restore contact inhibition. Moreover, we identified the C2 domain as an integral part of RasGAP’s catalytic unit, highlighting its importance for GAP activity.

That both merlin and RasGAP are required for proper Ras activity regulation, together with the identified interactions, points to a functional link between merlin and RasGAP. However, merlin appears not to directly regulate RasGAP’s catalytic activity. Given that the SH2-SH3-SH2 domains mediate RasGAP translocation from the cytoplasma to the plasma membrane through binding the consensus phosphotyrosine in various receptor tyrosine kinases (RTKs) [9], it is plausible that merlin acts in concert with activated RTKs to recruit RasGAP to fine-tune spatial-temporal Ras-GTP inactivation (Fig. 7d). Alternatively, merlin might recruit RasGAP independent of RTKs to suppress RTK-independent Ras activation and signaling. Note that merlin might not simultaneously bind to both Ras and RasGAP—these interactions might be highly transient and how they are regulated in vivo is not addressed in this study. The presence of merlin appears to modulate the signaling ability of Ras; however, the molecular details are still under investigation.

While RasGAP is a well-established negative regulator of Ras, our evidence does not support RasGAP as a prominent tumor suppressor. This is in agreement with genetic data that germline mutations of *RASA1* in humans cause the capillary malformation-arteriovenous malformation syndrome and other related vascular anomalies, without clear evidence for tumor predisposition [45]. Emerging evidence suggests that RasGAP is a weak tumor suppressor, whose loss contributes to tumor development in cooperation with additional factors [46–48].

An increasing body of literature suggests that merlin potentially suppresses Ras signaling at multiple levels, from RTKs to downstream. Merlin can suppress EGFR signaling during contact inhibition [49] and merlin loss leads to increased expression of multiple RTKs, including PDGFRβ [2]. Merlin can interfere with the formation of the Ras-activating Grb2/SOS/ezrin complex [5] and may also interact with Grb2 [50, 51]. Merlin loss leads to increased Ras expression at transcription level, mediated by YAP activation [52]. Downstream of Ras, overexpressed merlin can disrupt the B-Raf/Raf1/MLK3 complex formation by interacting with MLK3 [53]; merlin loss causes KSR1 overexpression and merlin may interact with KSR1 to interfere with KSR1:Raf1 interaction [54]. Our work suggests that merlin also acts at the Ras level. Notably, a merlin:RasGAP complex upstream of Ras was discovered; in addition, merlin targets Ras effector pathways, particularly the MEK1/2–Erk1/2 branch.

Finally, it is worth noting that merlin is involved in multiple pathways apart from Ras regulation and that its tumor suppressor activity is most likely a collective effect. However, given the extraordinarily high prevalence of Ras pathway activation in human tumors, including Schwann cell-derived tumors, e.g., neurofibromas caused by loss of *NF1* [4], regulation of Ras signaling very likely contributes significantly to merlin’s tumor suppressor activity.

## Materials and methods

### Cell culture

Sc4 is an immortalized Schwann cell line [5] established from *Nf2*^Δex2/Δex3^ mice by Marco Giovannini laboratory. RT4-D6P2T (RT4; subcloned from an ethylnitrosourea-induced rat schwannoma line [55]) was from European Collection of Authenticated Cell Cultures. RT4/Tet-NF2 (WT, S518A, or S518D) are derivatives of RT4, harboring doxycycline-inducible *NF2* transgenes [56]. *Rasa1*^−/−^ and *Rasa1*^+/+^ MEFs [39] were from Christian Widmann, originally from Tony Pawson. 293 and 293T cells were from ATCC. The above-mentioned lines were grown in DMEM/10% FBS. *Nf2*^+/+^ (FH-912, genotype *Nf2*^flox2/flox2^) and *Nf2*^−/−^ (FC-1801, genotype *Nf2*^Δex2/Δex2^) mSCs [10] were from Marco Giovannini. MSCs and the derivatives were grown in DMEM:F12-HAM (1:1) medium plus N2 supplement (Gibco), 2% FBS, 1 μM forskolin, and 10 ng/ml heregulin-β1 (recombinant EGF-like domain; PeproTech), on noncoated tissue culture plates. Or, plates were coated with 0.05 mg/ml poly-L-lysine (Sigma-Aldrich), air dried, and further coated with DMEM/10% FBS; the cells were grown in the above-mentioned medium omitting FBS. All cells were maintained in a humidified atmosphere with 5% CO_2_ at 37 °C.

### Orthotopic transplantation and bioluminescence imaging

All animal experiments were approved by the Wistar Institutional Animal Care and Use Committee and performed in accordance with relevant institutional and national guidelines. *Nf2*^+/+^ mSCs were first transduced with lentiviruses carrying pLU-Tet-EF1aL-FFluc-mCherry (encoding firefly luciferase and mCherry, provided by the Wistar Institute Vector Core), and sorted by FACS. The established luciferase-expressing mSCs/Luc were further transduced with the KD lentiviruses and selected with puromycin. A total of 2 × 10^5^ cells were transplanted into the sciatic nerve sheath (one side) of each NOD/SCID mouse (Jackson labs stock# 005557; male, 6–8 weeks of age; *n* = 8–9 per group) by intraneural injection. Tumor growth was monitored weekly for a total of 3 weeks by bioluminescence imaging on an IVIS-200 system, according to the manufacturer’s instructions (Xenogen, San Francisco, CA).

### Statistical analysis

Statistical analyses were performed with GraphPad Prism 7.

## Supplementary information

The combined SI File

## References

[1] Evans DG (2009). Neurofibromatosis 2 [bilateral acoustic neurofibromatosis, central neurofibromatosis, NF2, neurofibromatosis type II]. Genet Med.

[2] Petrilli AM, Fernandez-Valle C (2016). Role of merlin/NF2 inactivation in tumor biology. Oncogene.

[3] Clucas J, Valderrama F (2014). ERM proteins in cancer progression. J Cell Sci.

[4] Simanshu DK, Nissley DV, McCormick F (2017). RAS proteins and their regulators in human disease. Cell.

[5] Morrison H, Sperka T, Manent J, Giovannini M, Ponta H, Herrlich P (2007). Merlin/neurofibromatosis type 2 suppresses growth by inhibiting the activation of Ras and Rac. Cancer Res.

[6] Ammoun S, Flaiz C, Ristic N, Schuldt J, Hanemann CO (2008). Dissecting and targeting the growth factor-dependent and growth factor-independent extracellular signal-regulated kinase pathway in human schwannoma. Cancer Res.

[7] Tikoo A, Varga M, Ramesh V, Gusella J, Maruta H (1994). An anti-Ras function of neurofibromatosis type 2 gene product (NF2/Merlin). J Biol Chem.

[8] Hirokawa Y, Tikoo A, Huynh J, Utermark T, Hanemann CO, Giovannini M (2004). A clue to the therapy of neurofibromatosis type 2: NF2/merlin is a PAK1 inhibitor. Cancer J.

[9] Maertens O, Cichowski K (2014). An expanding role for RAS GTPase activating proteins (RAS GAPs) in cancer. Adv Biol Regul.

[10] Li W, You L, Cooper J, Schiavon G, Pepe-Caprio A, Zhou L (2010). Merlin/NF2 suppresses tumorigenesis by inhibiting the E3 ubiquitin ligase CRL4(DCAF1) in the nucleus. Cell.

[11] Meier C, Parmantier E, Brennan A, Mirsky R, Jessen KR (1999). Developing Schwann cells acquire the ability to survive without axons by establishing an autocrine circuit involving insulin-like growth factor, neurotrophin-3, and platelet-derived growth factor-BB. J Neurosci.

[12] Lobsiger CS, Schweitzer B, Taylor V, Suter U (2000). Platelet-derived growth factor-BB supports the survival of cultured rat Schwann cell precursors in synergy with neurotrophin-3. Glia.

[13] Monje PV, Rendon S, Athauda G, Bates M, Wood PM, Bunge MB (2009). Non-antagonistic relationship between mitogenic factors and cAMP in adult Schwann cell re-differentiation. Glia.

[14] Bondeva T, Balla A, Varnai P, Balla T (2002). Structural determinants of Ras-Raf interaction analyzed in live cells. Mol Biol Cell.

[15] Sperka T, Geissler KJ, Merkel U, Scholl I, Rubio I, Herrlich P (2011). Activation of Ras requires the ERM-dependent link of actin to the plasma membrane. PLoS ONE.

[16] Geissler KJ, Jung MJ, Riecken LB, Sperka T, Cui Y, Schacke S (2013). Regulation of Son of Sevenless by the membrane-actin linker protein ezrin. Proc Natl Acad Sci USA.

[17] Pearson MA, Reczek D, Bretscher A, Karplus PA (2000). Structure of the ERM protein moesin reveals the FERM domain fold masked by an extended actin binding tail domain. Cell.

[18] Riecken LB, Tawamie H, Dornblut C, Buchert R, Ismayel A, Schulz A (2015). Inhibition of RAS activation due to a homozygous ezrin variant in patients with profound intellectual disability. Hum Mutat.

[19] Newlaczyl AU, Coulson JM, Prior IA (2017). Quantification of spatiotemporal patterns of Ras isoform expression during development. Sci Rep.

[20] Mo SP, Coulson JM, Prior IA (2018). RAS variant signalling. Biochem Soc Trans.

[21] Kim DI, Jensen SC, Noble KA, Kc B, Roux KH, Motamedchaboki K (2016). An improved smaller biotin ligase for BioID proximity labeling. Mol Biol Cell.

[22] Hoshino M, Kawakita M, Hattori S (1988). Characterization of a factor that stimulates hydrolysis of GTP bound to ras gene productp21 (GTPase-activating protein) and correlation of its activity to cell density. Mol Cell Biol.

[23] Shyu YJ, Liu H, Deng X, Hu CD (2006). Identification of new fluorescent protein fragments for bimolecular fluorescence complementation analysis under physiological conditions. Biotechniques.

[24] Orthaus S, Klement K, Happel N, Hoischen C, Diekmann S (2009). Linker histone H1 is present in centromeric chromatin of living human cells next to inner kinetochore proteins. Nucleic Acids Res.

[25] Cole BK, Curto M, Chan AW, McClatchey AI (2008). Localization to the cortical cytoskeleton is necessary for Nf2/merlin-dependent epidermal growth factor receptor silencing. Mol Cell Biol.

[26] Li Y, Zhou H, Li F, Chan SW, Lin Z, Wei Z (2015). Angiomotin binding-induced activation of Merlin/NF2 in the Hippo pathway. Cell Res.

[27] Eerola I, Boon LM, Mulliken JB, Burrows PE, Dompmartin A, Watanabe S (2003). Capillary malformation-arteriovenous malformation, a new clinical and genetic disorder caused by RASA1 mutations. Am J Hum Genet.

[28] Gusella JF, Ramesh V, MacCollin M, Jacoby LB (1999). Merlin: the neurofibromatosis 2 tumor suppressor. Biochim Biophys Acta.

[29] Sher I, Hanemann CO, Karplus PA, Bretscher A (2012). The tumor suppressor merlin controls growth in its open state, and phosphorylation converts it to a less-active more-closed state. Dev Cell.

[30] Li Q, Nance MR, Kulikauskas R, Nyberg K, Fehon R, Karplus PA (2007). Self-masking in an intact ERM-merlin protein: an active role for the central alpha-helical domain. J Mol Biol.

[31] Hennigan RF, Foster LA, Chaiken MF, Mani T, Gomes MM, Herr AB (2010). Fluorescence resonance energy transfer analysis of merlin conformational changes. Mol Cell Biol.

[32] Chinthalapudi K, Mandati V, Zheng J, Sharff AJ, Bricogne G, Griffin PR (2018). Lipid binding promotes the open conformation and tumor-suppressive activity of neurofibromin 2. Nat Commun.

[33] Fehon RG, McClatchey AI, Bretscher A (2010). Organizing the cell cortex: the role of ERM proteins. Nat Rev Mol Cell Biol.

[34] Ahronowitz I, Xin W, Kiely R, Sims K, MacCollin M, Nunes FP (2007). Mutational spectrum of the NF2 gene: a meta-analysis of 12 years of research and diagnostic laboratory findings. Hum Mutat.

[35] Kang BS, Cooper DR, Devedjiev Y, Derewenda U, Derewenda ZS (2002). The structure of the FERM domain of merlin, the neurofibromatosis type 2 gene product. Acta Crystallogr D Biol Crystallogr.

[36] Shimizu T, Seto A, Maita N, Hamada K, Tsukita S, Tsukita S (2002). Structural basis for neurofibromatosis type 2. Crystal structure of the merlin FERM domain. J Biol Chem.

[37] Drugan JK, Rogers-Graham K, Gilmer T, Campbell S, Clark GJ (2000). The Ras/p120 GTPase-activating protein (GAP) interaction is regulated by the p120 GAP pleckstrin homology domain. J Biol Chem.

[38] Gideon P, John J, Frech M, Lautwein A, Clark R, Scheffler JE (1992). Mutational and kinetic analyses of the GTPase-activating protein (GAP)-p21 interaction: the C-terminal domain of GAP is not sufficient for full activity. Mol Cell Biol.

[39] van der Geer P, Henkemeyer M, Jacks T, Pawson T (1997). Aberrant Ras regulation and reduced p190 tyrosine phosphorylation in cells lacking p120-Gap. Mol Cell Biol.

[40] Koehler JA, Moran MF (2001). Regulation of extracellular signal-regulated kinase activity by p120 RasGAP does not involve its pleckstrin homology or calcium-dependent lipid binding domains but does require these domains to regulate cell proliferation. Cell Growth Differ.

[41] Shin SI, Freedman VH, Risser R, Pollack R (1975). Tumorigenicity of virus-transformed cells in nude mice is correlated specifically with anchorage independent growth in vitro. Proc Natl Acad Sci USA.

[42] Colburn NH, Bruegge WF, Bates JR, Gray RH, Rossen JD, Kelsey WH (1978). Correlation of anchorage-independent growth with tumorigenicity of chemically transformed mouse epidermal cells. Cancer Res.

[43] Lin TH, Chen Q, Howe A, Juliano RL (1997). Cell anchorage permits efficient signal transduction between ras and its downstream kinases. J Biol Chem.

[44] Loza-Coll MA, Perera S, Shi W, Filmus J (2005). A transient increase in the activity of Src-family kinases induced by cell detachment delays anoikis of intestinal epithelial cells. Oncogene.

[45] Bayrak-Toydemir P, Stevenson D. RASA1-related disorders. In: Adam MP, Ardinger HH, Pagon RA, Wallace SE, Bean LJH, Stephens K, Amemiya A, editors. GeneReviews® [Internet]. Seattle (WA): University of Washington, Seattle; 1993–2019.

[46] Lubeck BA, Lapinski PE, Oliver JA, Ksionda O, Parada LF, Zhu Y (2015). Cutting edge: codeletion of the Ras GTPase-activating proteins (RasGAPs) neurofibromin 1 and p120 RasGAP in T cells results in the development of T cell acute lymphoblastic leukemia. J Immunol.

[47] Suarez-Cabrera C, Quintana RM, Bravo A, Casanova ML, Page A, Alameda JP (2017). A transposon-based analysis reveals RASA1 is involved in triple-negative breast cancer. Cancer Res.

[48] Hayashi T, Desmeules P, Smith RS, Drilon A, Somwar R, Ladanyi M (2018). RASA1 and NF1 are preferentially co-mutated and define a distinct genetic subset of smoking-associated non-small cell lung carcinomas sensitive to MEK inhibition. Clin Cancer Res.

[49] Curto M, Cole BK, Lallemand D, Liu CH, McClatchey AI (2007). Contact-dependent inhibition of EGFR signaling by Nf2/Merlin. J Cell Biol.

[50] Wiederhold T, Lee MF, James M, Neujahr R, Smith N, Murthy A (2004). Magicin, a novel cytoskeletal protein associates with the NF2 tumor suppressor merlin and Grb2. Oncogene.

[51] Lim JY, Kim H, Jeun SS, Kang SG, Lee KJ (2006). Merlin inhibits growth hormone-regulated Raf-ERKs pathways by binding to Grb2 protein. Biochem Biophys Res Commun.

[52] Garcia-Rendueles ME, Ricarte-Filho JC, Untch BR, Landa I, Knauf JA, Voza F (2015). NF2 Loss promotes oncogenic RAS-induced thyroid cancers via YAP-dependent transactivation of RAS proteins and sensitizes them to MEK inhibition. Cancer Discov.

[53] Chadee DN, Xu D, Hung G, Andalibi A, Lim DJ, Luo Z (2006). Mixed-lineage kinase 3 regulates B-Raf through maintenance of the B-Raf/Raf-1 complex and inhibition by the NF2 tumor suppressor protein. Proc Natl Acad Sci USA.

[54] Zhou L, Lyons-Rimmer J, Ammoun S, Muller J, Lasonder E, Sharma V (2016). The scaffold protein KSR1, a novel therapeutic target for the treatment of merlin-deficient tumors. Oncogene.

[55] Bansal R, Pfeiffer SE (1987). Regulated galactolipid synthesis and cell surface expression in Schwann cell line D6P2T. J Neurochem.

[56] Morrison H, Sherman LS, Legg J, Banine F, Isacke C, Haipek CA (2001). The NF2 tumor suppressor gene product, merlin, mediates contact inhibition of growth through interactions with CD44. Genes Dev.

[57] Yang JY, Widmann C (2001). Antiapoptotic signaling generated by caspase-induced cleavage of RasGAP. Mol Cell Biol.

[58] Scheffzek K, Ahmadian MR, Kabsch W, Wiesmuller L, Lautwein A, Schmitz F (1997). The Ras-RasGAP complex: structural basis for GTPase activation and its loss in oncogenic Ras mutants. Science.

