# Supplementary material for: The NF2 tumor suppressor merlin interacts with Ras and RasGAP, which may modulate Ras signaling
Source: Oncogene. 2019 Jul 16;38(36):6370–81. doi: 10.1038/s41388-019-0883-6 (PMC6756068; doi:10.1038/s41388-019-0883-6)
Supplement: Supplementary file 1 — The combined SI File [file 41388_2019_883_MOESM1_ESM.pdf]

Supplementary Materials for

The *NF2* tumor suppressor merlin interacts with Ras and RasGAP, which may modulate Ras signaling

Yan Cui, Susann Groth, Scott Troutman, Annemarie Carlstedt, Tobias Sperka, Lars Björn Riecken, Joseph L. Kissil, Hongchuan Jin, Helen Morrison

This PDF file includes:

Figs. S1 to S9

Supplementary Materials and Methods

Supplementary References

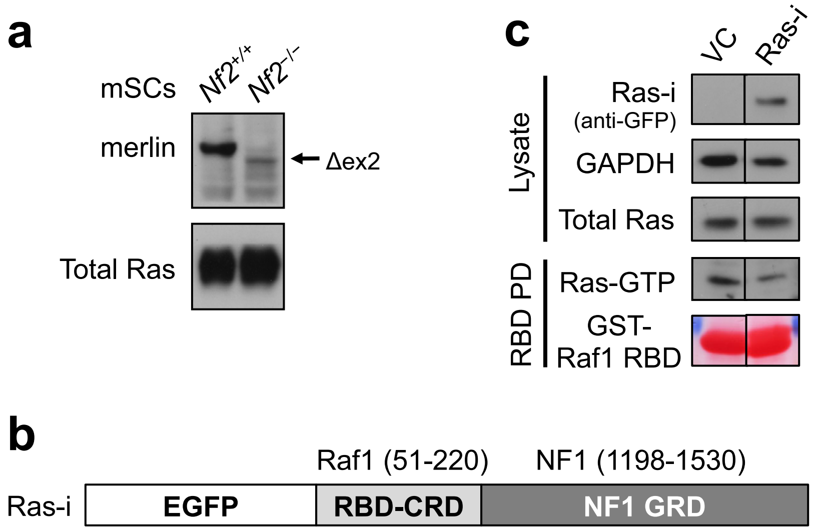

Fig. S1

Validation of *Nf2*$^{-/-}$ and *Nf2*$^{+/+}$ mouse Schwann cells (mSCs) and Ras-i functionality. **a** *Nf2* exon 2 knockout (Δex2) in mSCs was validated by Western blotting. **b** The artificial Ras inhibitory protein (Ras-i) consists of three fused protein fragments as illustrated. Amino acid positions in the related full length proteins (human version) are indicated. **c** Expression of Ras-i in *Nf2*$^{-/-}$ mSCs resulted in decreased Ras-GTP level, analyzed by GST-Raf1 RBD pulldown and subsequent Western blotting. The same samples as in Fig. 1c, d were used. Spliced lanes are from the same exposure of the same blot.

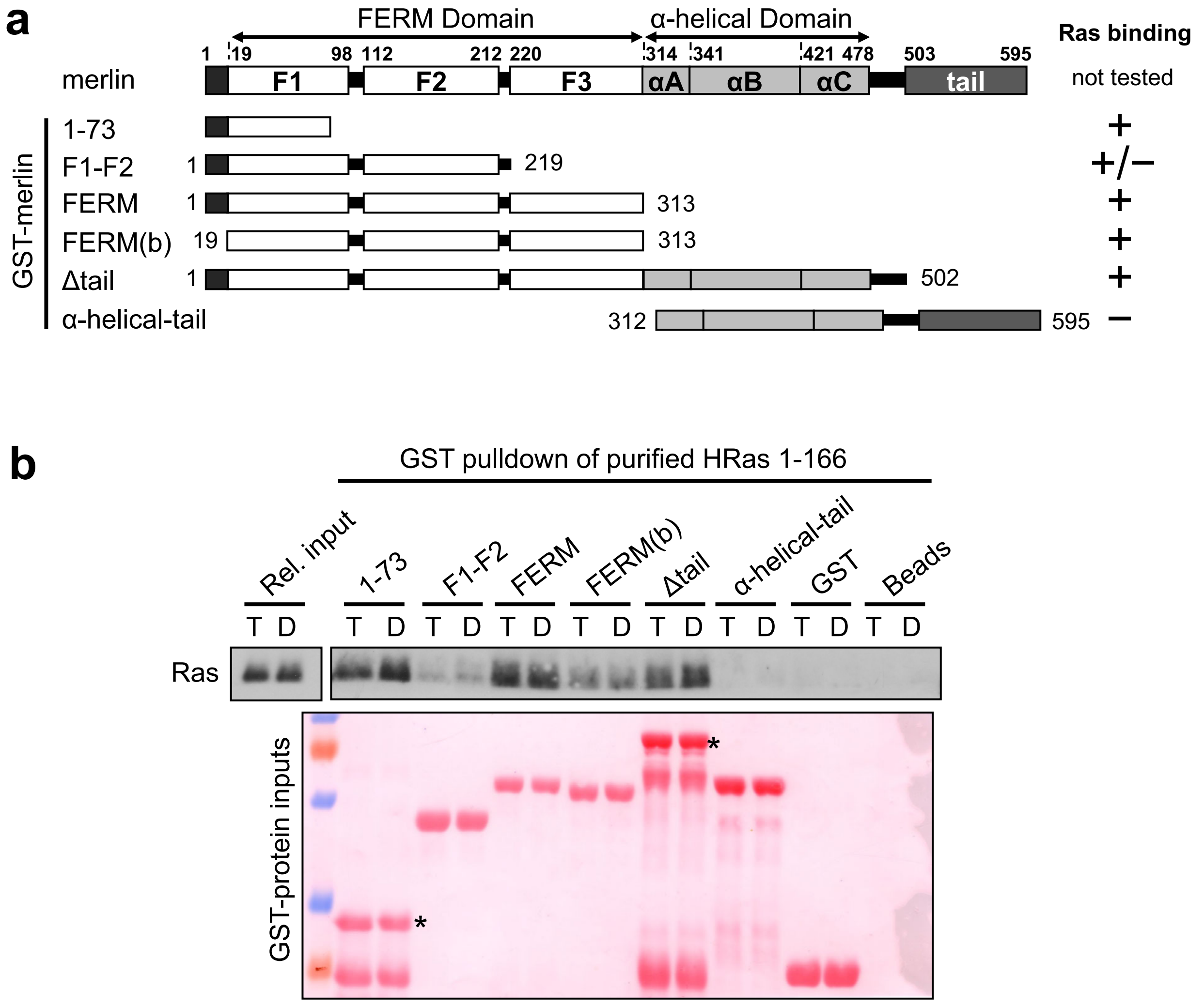

Fig. S2

The F1 subdomain of merlin can directly bind Ras. **a** Schematic of domain organization of merlin and the GST-fusion fragments used for the pulldown experiments. **b** Western blot analysis of GST-merlin fragments pulldown of HRas 1–166 preloaded with GDP (D) or GTPγS (T) . All proteins were purified from *E. coli*. GST-fusion protein inputs were stained with Ponceau S. Asterisks indicate correct bands in case of multiple bands. Note that both the FERM(b) and the merlin F1-F2 fragments showed greatly reduced binding to Ras, likely an effect of conformational change caused by the additional deletions.

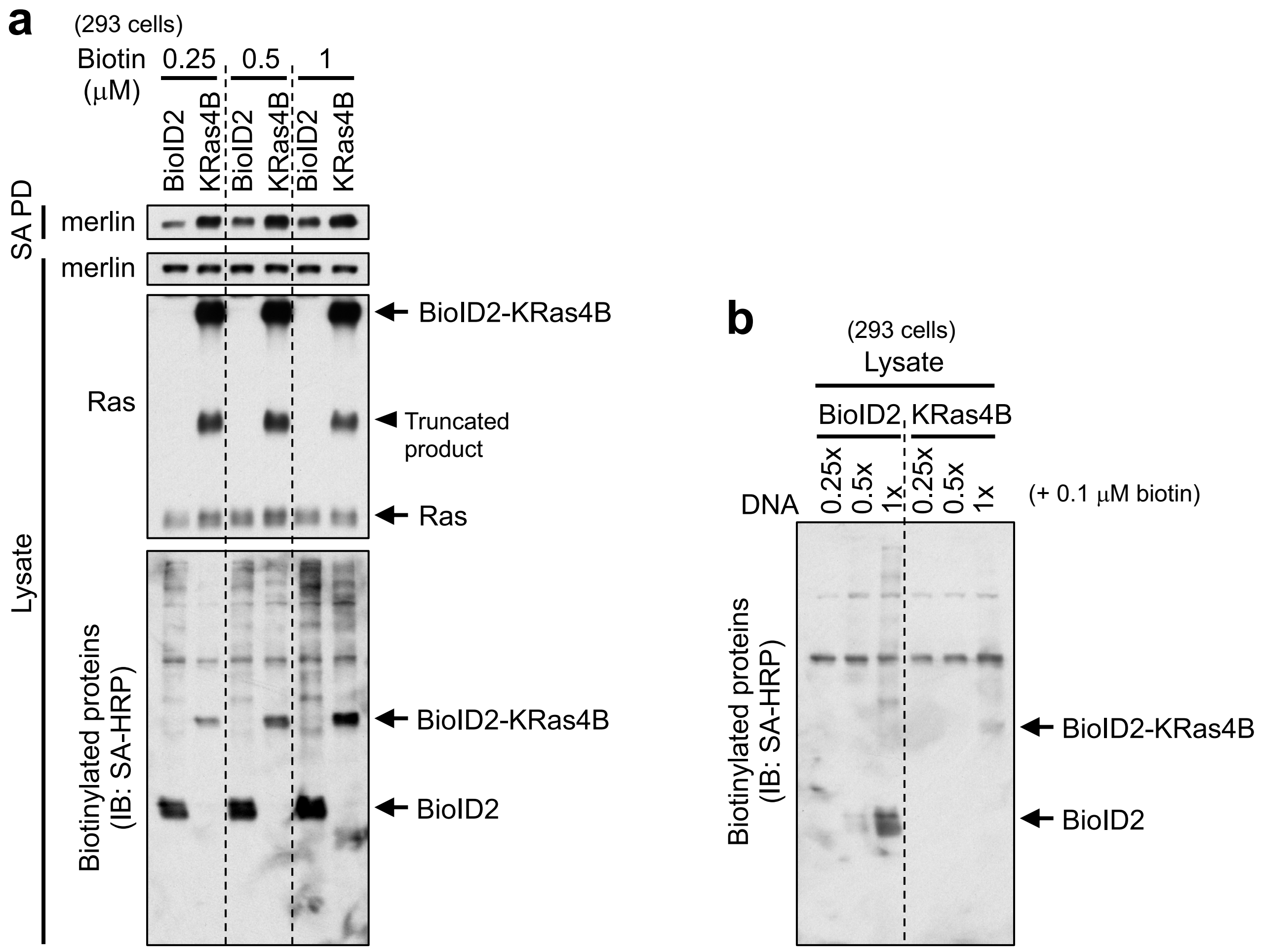

Fig. S3

In vivo interaction between merlin and KRas4B demonstrated by proximity biotinylation, related to Fig. 2. **a** Indicated constructs (with an N-terminal OLLAS tag) were transfected into 293 cells; ~24 h later, media was replenished with biotin added; ~48 h after transfection, cells were lysed for Streptavidin (SA) pulldown (PD), analyzed by Western blotting. Note that BioID2-KRas4B biotinylated merlin more efficiently than BioID2, even though BioID2 alone exhibited much higher non-specific biotinylation activity. **b** Overall biotinylation activity assessment for Fig. 2d, as described above. IB: immunoblotting.

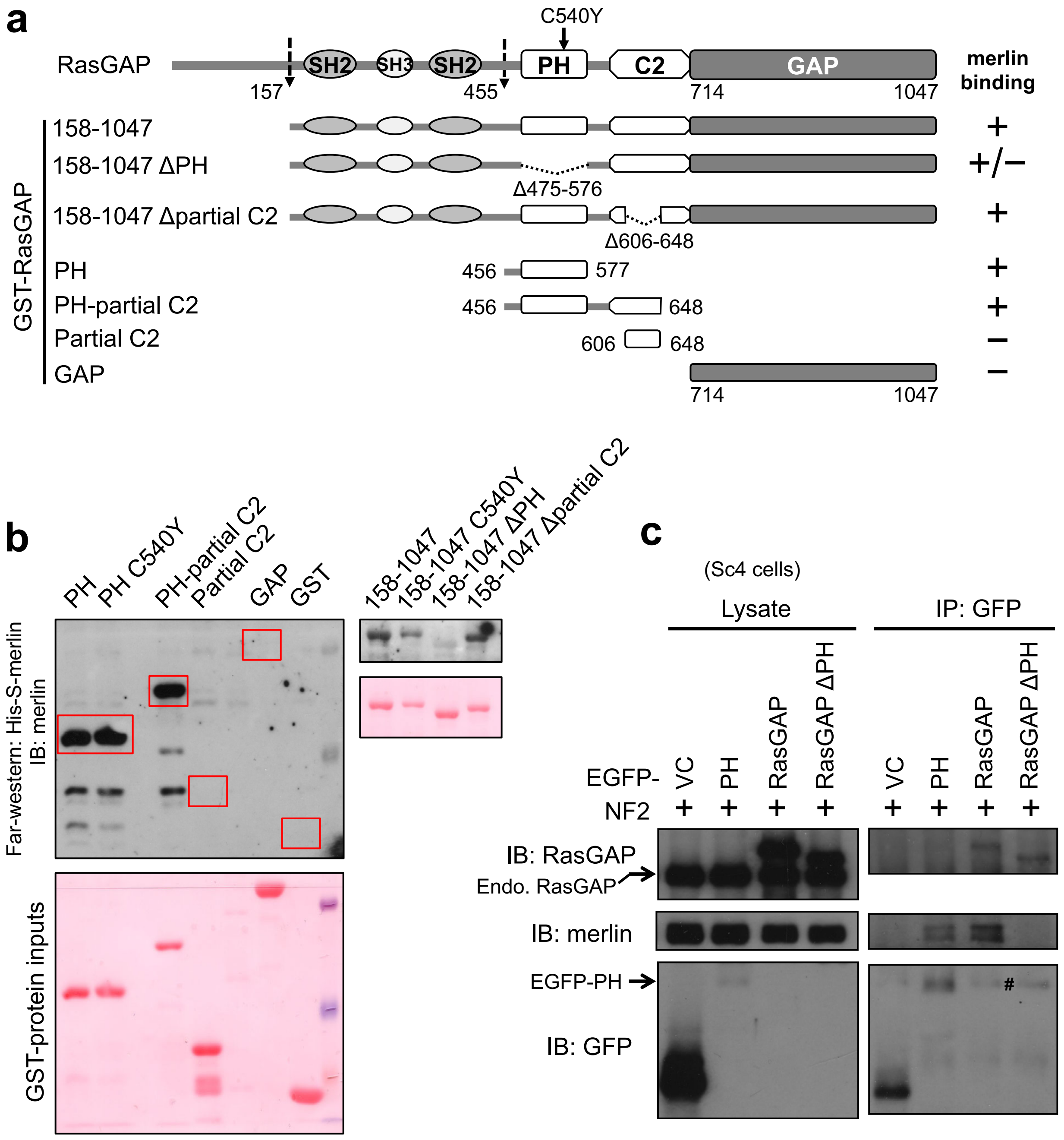

Fig. S4

The PH domain of RasGAP is the main site for interacting with merlin. **a** Schematic of domain organization of RasGAP and the GST-RasGAP fragments used as baits in the Far-western blot analysis. **b** Far-western blot analysis of merlin:RasGAP interaction. All the proteins were purified from *E. coli*. The baits were separated by SDS-PAGE and blotted onto Nitrocellulose-membrane. The membrane was blocked with milk and incubated with His-S-tagged merlin. The bound merlin was detected by Western blotting using NF2 (B-12) antibody. The baits were detected by Ponceau S staining. The red boxes indicate the locations of the baits. **c** Western blot analysis of Co-IP of merlin with EGFP-RasGAP fragments. The constructs were co-transfected into Sc4 cells; IP was performed with GFP (FL) antibody (polyclonal). # indicates an interfering band from the antibody for IP, running at the same size as EGFP-PH. IB: immunoblotting.

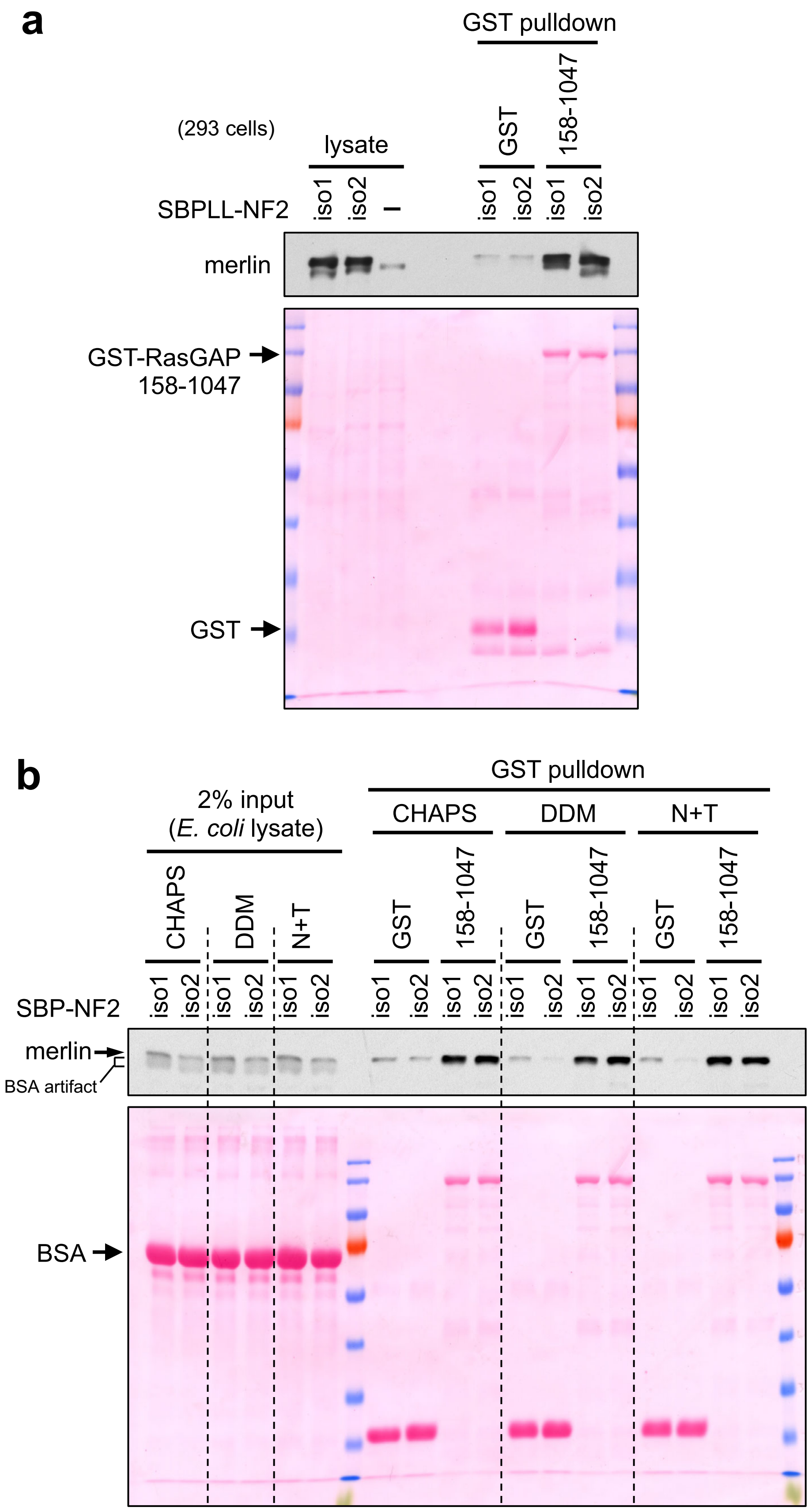

Fig. S5

RasGAP can also bind merlin isoform 2. **a, b** Western blot analysis of GST-RasGAP (aa 157–1047) pulldown of SBPLL-merlin isoforms (S518A) overexpressed in 293 by transient transfection (**a**) or SBP-merlin isoforms expressed in *E. coli* (**b**). GST-fusion protein inputs were stained with Ponceau S. For binding, the N+T buffer was used in **a**, whereas three buffers with 0.1% BSA in **b**. Note that the concentration of merlin iso2 was lower than that of iso1 in **b**. N+T: 2% NOG + 1% Triton X-100.

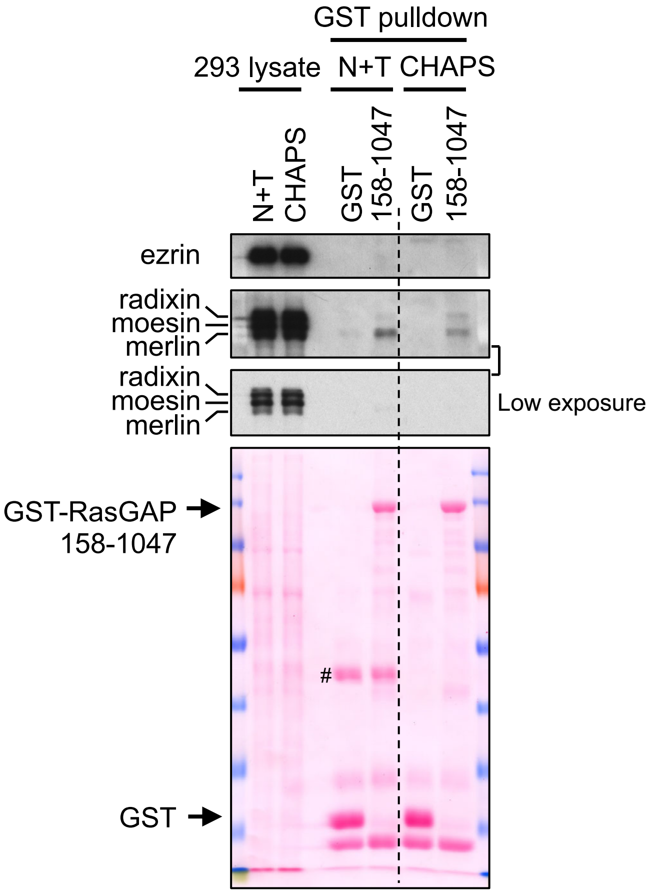

Fig. S6
RasGAP may prefer to bind merlin rather than ERMs. Western blot analysis of GST-RasGAP (aa 157–1047) pulldown of endogenous ERMs and merlin from 293 lysate. GST-fusion protein inputs were stained with Ponceau S. Ezrin was probed first with the 3C12 antibody (mouse monoclonal), with no specific signal detected in the pulldown. The membrane was treated with 30% H_2O_2 to inactivate the bound anti-mouse IgG-HRP; then it was co-incubated with three rabbit monoclonal antibodies against radixin, moesin, and merlin, respectively (our prior experiments have showed that only ezrin and radixin partially overlapped on SDS-PAGE, whereas the others could be separated). Moesin was non-detectable in the pulldown. Note that the signal from merlin was relatively stronger in the pulldown but weaker in the lysate, compared with those from radixin or moesin, suggesting more efficient binding to RasGAP by merlin, although the real expression levels of these proteins could not be inferred. # indicates a protein that strongly bound to glutathione sepharose under the N+T buffer condition, which was likely EF1 gamma, identified by mass spectrometry. Note that EF1 gamma contains a GST-like domain [13]; here it could serve as an additional control for sepharose inputs. N+T: 2% NOG + 1% Triton X-100.

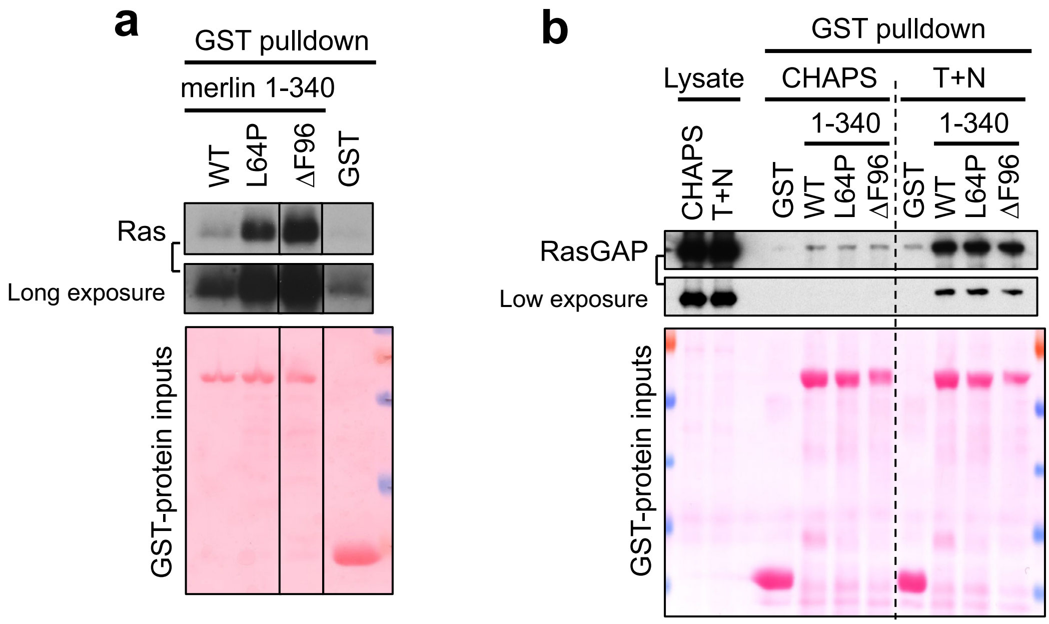

Fig. S7

Two NF2 patient-derived mutations do not impair merlin's binding to Ras or RasGAP *in vitro.* **a, b** Western blot analysis of GST-merlin (aa 1–340, WT and the mutants) pulldown of purified HRas 1–166 preloaded with GDP (**a**) or non-tagged full-length RasGAP overexpressed in 293 by transient transfection (**b**). GST-fusion protein inputs were stained with Ponceau S. The mutants showed increased binding to Ras, whereas they bound to RasGAP similarly to the WT. N+T: 2% NOG + 1% Triton X-100.

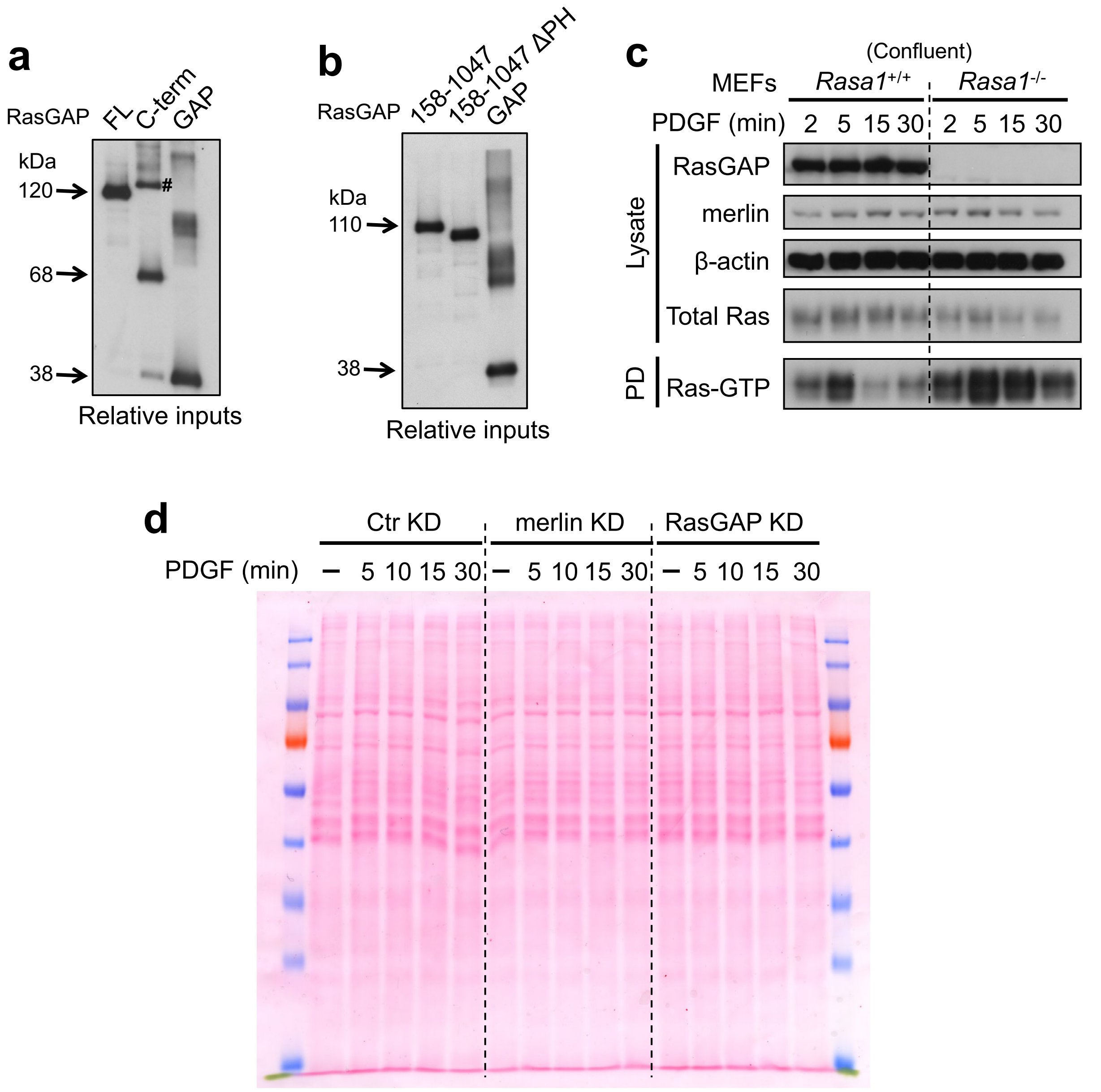

Fig. S8

a, **b** Related to Fig. 6b, c. Relative inputs were determined by Western blot analysis of RasGAP fragments from the same fresh dilution for the GAP activity experiments using a RasGAP (C-term) polyclonal antibody. # indicates an unknown product with a much bigger size. **c** Confluent *Rasa1*$^{+/+}$ and *Rasa1*$^{-/-}$ MEFs were serum starved overnight, stimulated with 10 ng/ml PDGF-BB and lysed at indicated time points for Ras-GTP pulldown by GST-Raf1 RBD, analyzed by Western blotting. **d** Related to Fig. 6d. Ponceau S staining of the whole membrane as loading control (prior to blocking).

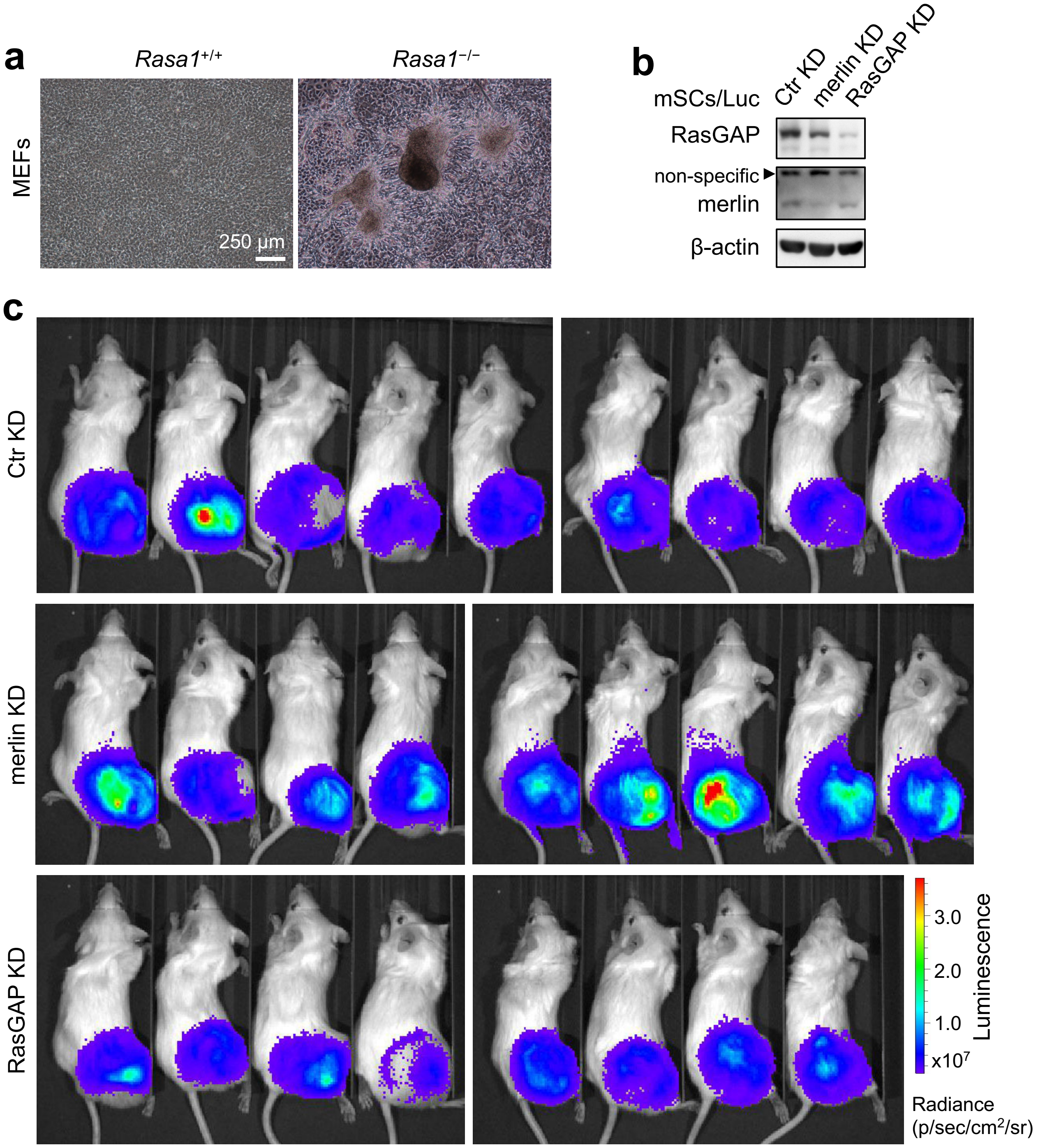

Fig. S9

Evaluation of loss of RasGAP in cellular transformation. **a** Focus formation in *Rasa1*$^{-/-}$ MEFs. Cells were seeded to near confluence, and maintained for 3–4 weeks. Growth medium was changed every 2–4 days. No foci were observed in *Rasa1*$^{+/+}$ MEFs. **b, c** Related to Fig. 7a, b. **b** Knockdown of merlin and RasGAP in mSCs/Luc was verified by Western blotting. **c** Endpoint luminescence images from all mice in Fig. 7b.

Supplementary Materials and Methods

Antibodies:

Antibody	Clone/ID	Cat. No.	Supplier	Application
Akt1/2/3	H-136 (poly)	sc-8312	Santa Cruz	IB
Erk 2	K-23 (poly)	sc-153	Santa Cruz	IB
ERK 1/2	C-9	sc-514302	Santa Cruz	IB
ezrin	3C12	MS-661-P1	NeoMarkers	IB
GAPDH	6C5	sc-32233	Santa Cruz	IB
GFP	B-2	sc-9996	Santa Cruz	IB
GFP	FL (poly)	sc-8334	Santa Cruz	IB, IP
MEK1/2	D1A5	8727	Cell Signaling	IB
MEK1/2	L38C12	4694	Cell Signaling	IB
moesin	EPR2428(2)	ab151542	abcam	IB
merlin	D3S3W	12888	Cell Signaling	IB
merlin	D6N8H	12896	Cell Signaling	IB
NF2	B-12	sc-55575	Santa Cruz	IB
NF2	A-19 (poly)	sc-331	Santa Cruz	IB, IP
NF2	AF1G4	ab88957	abcam	IB
OLLAS Tag	L2	MA5-16125	Invitrogen	IB
p-Akt (T308)	D25E6 (XP)	13038	Cell Signaling	IB
p-Akt (S473)	D9E (XP)	4060	Cell Signaling	IB
p-c-Raf (S338)	56A6	9427	Cell Signaling	IB
PDGFRβ	28E1	3169	Cell Signaling	IB
p-Erk1/2 (T202/Y204)	D13.14.4E (XP)	4370	Cell Signaling	IB
p-MEK1/2 (S217/221)	41G9	9154	Cell Signaling	IB
p-PDGFRβ (Y857)	J24-425	558360	BD Biosciences	IB
radixin	EP1862Y	ab52495	abcam	IB
Raf-1	C-12 (poly)	sc-133	Santa Cruz	IB
Ras	(From the Kit)	16117	Pierce	IB
Ras	Ras10	MA1-012	Invitrogen	IB
Ras	EP1125Y	ab52939	abcam	IB
Ras GAP	B4F8	sc-63	Santa Cruz	IB
RasGAP	13/RAS-GAP	610040	BD Biosciences	IB
RasGAP	C-term (poly)	1715-1	Epitomics	IB
β-actin	AC-15	A5441	Sigma-Aldrich	IB
normal rabbit IgG		sc-2027	Santa Cruz	IP
anti-Rabbit IgG-HRP		P0448	Dako	IB
anti-Mouse IgG-HRP		P0447	Dako	IB
anti-Rat IgG-HRP		61-9520	Invitrogen	IB
Streptavidin-HRP		21130	Pierce	IB
anti-Mouse IgG-HRP		ab97040	Abcam	IB
anti-Rabbit IgG-HRP		ab97080	Abcam	IB

IB: immunoblotting; IP: immunoprecipitation.

Plasmids

Throughout this manuscript, NF2 and merlin may be used interchangeably. All the NF2, RasGAP, Raf1 and NF1 cDNA, full-length or fragments, are of human origin. General techniques in molecular cloning and mutagenesis were used; all the constructs were verified by restriction digestion and/or DNA sequencing.

pcDNA3-Flag-NF2 (iso1) was from David Gutmann (Washington University School of Medicine, USA). h-RasGAP.dn3 [1] was from Christian Widman (University of Lausanne, Switzerland). pGEX-4T-1-GAP (aa 714–1047) and pGEX-4T-1-NF1 GRD (aa 1198–1530 of the type 1 isoform) were from Reza Ahmadian (Medical Faculty of the Heinrich-Heine University, Germany). The BiFC vectors pCerf-C2 and pCerl-C2 (aa 1–172 and aa 154–238 of Cerulean, respectively, not counting the start codon) [2], were from Christian Hoischen (Leibniz Institute on Aging – FLI, Germany). pET15TEV-HRas 1–166 [3] and pGEX-Raf1 RBD (aa 51–131) [4] have been described before. pGEX-BRaf RBD (aa 150–233) [5] was from Mitsu Ikura & Matthew J. Smith. pDONR223-RAF1 (Addgene #23832) [6] was from William Hahn & David Root. The lentiviral packaging plasmids psPAX2 (Addgene #12260) and pCMVΔR8.91 [7], and the VSV-G envelope plasmid pMD2.G (Addgene #12259) were from Didier Trono. pCMV-VSV-G (Addgene #8454) [8] was from Bob Weinberg.

The lentiviral vector pCDH-CMV.Bsd was generated by replacing the puromycin selection marker in pCDH-CMV-MCS-EF1-puro (System Biosciences) with blasticidin selection marker BSD, in two steps. First, the site upstream of the puromycin resistant gene was mutated into Avr II site. Second, the puromycin resistant gene was digested out with Avr II and Sal I; *Bsd* ORF was PCR amplified using a pcDNA6 vector (Invitrogen) as the template, digested and ligated into the two sites.

Lentiviral vectors with an SBP tag or without a tag were constructed as described in [9]. We later also constructed vectors with a long linker (LL) following the SBP-tag (SBPLL) or with an OLLAS tag. BioID2 cDNA [10] was amplified by PCR and cloned into the lentiviral vector with an EFS promoter and an OLLAS tag (pCDH-EFS-OLLAS).

pCDH-CMV-EGFP-CY.Bsd was generated in two steps. To begin, the Nhe I–BamH I fragment (containing EGFP ORF) from pEGFP-C2 (Clontech) vector was ligated into the same sites of pCDH-CMV.Bsd; the resultant vector was named pCDH-CMV-EGFP-C2.Bsd. Next, the BsrG I–Not I fragment (the MCS region) of pCDH-CMV-EGFP-C2.Bsd was replaced by a customized MCS.

pCDH-CMV-EGFP-Raf1 (51–220)-NF1 (GRD).Bsd was generated in two steps. Initially, Raf1 (51–220) was amplified by PCR using pDONR223-RAF1 as template, digested and inserted into BamH I and EcoR I sites of pCDH-CMV-EGFP-CY.Bsd; the resultant vector was named pCDH-CMV-EGFP-Raf1 (51–220)ns (ns: no stop codon). To follow, the EcoR I–Not I fragment from pGEX-4T-1-NF1 GRD was ligated into the same sites of pCDH-CMV-EGFP-Raf1 (51–220)ns to obtain the final construct.

NF2 iso1 ORF with BamH I and Not I sites added at the 5' and the 3' end respectively, was amplified by PCR and cloned into pCR4Blunt-TOPO (Invitrogen). Subsequently, the ORF was subcloned into pGEX-4T-1 (GE Healthcare), pET30a (+) (Novagen), pcDNA3.1 (+) (Invitrogen) and pCDH-CMV.Bsd using the BamH I and Not I sites, and into pCerf-C2 using the EcoR I site.

NF2 iso2 tail with BamH I and Not I sites added was amplified by PCR and cloned into pGEX-4T-1. *NF2* iso2 full-length ORF was re-assembled using iso1 ORF and a 3' fragment from iso2, and cloned into vectors for mammalian or bacterial expression.

KRas4B cDNA (from Ion C. Cirstea) was subcloned into pcDNA3.1(+) and also the lentiviral EFS-OLLAS-BioID2 vector.

pcDNA3/GAP was modified from h-RasGAP.dn3 by mutagenesis to delete the partial 5' and 3' UTR and to change the 3' EcoR I into BamH I. Subsequently, RasGAP ORF was sunbcloned into pGEX-4T-1, pEGFP-C2, pCerl-C2, and the lentiviral vector with an *EF1a* promoter using the 5' EcoR I site and an appropriate 3' site. All the deletion and point mutation mutants were generated by either mutagenesis or PCR cloning.

For expression of SBP-tagged proteins in *E. coli*, SBP–MCS (with Nde I and Sal I sites added at the respective end) was amplified by PCR, using the lentiviral SBP vector as

template and digested to replace the Nde I–Xho I fragment of pET31b (Novagen); the resultant vector was named pET-A-SBP. Likewise, we later constructed pET-A-SBPLL. *NF2* iso1 and 2 cDNAs were subcloned into pET-A-SBP. RasGAP fragments were subcloned into pET-A-SBPLL.

For lentiviral knockdown (KD) we have constructed a vector named pLV-H1-GIPZ, modified from pGIPZ (Open Biosystems) to enable improved and more versatile KD. The targeting sequences embedded in an optimized miRNA scaffold were cloned into pLV-H1-GIPZ. The artificial miRNA(s) is under a CMV promoter, embedded in a big transcript. The RasGAP-KD vector also contains a second targeting sequence in a conventional shRNA under the H1 promoter; the combination of an shRNA and a miRNA in the same vector further improved the KD efficiency (data not shown). To further improve the KD efficiency for RasGAP, we constructed the pLV-U6-GIPZ vector, and placed the shRNA under the U6 promoter in combination with the miRNA; this U6-version vector was only used in Fig. 7c. Details of the design will be published elsewhere. The NF2 target sequence was *gtgacaaggagtttactattaa* (miR); the RasGAP target sequences were *tcaggtcagcagccttgtttta* (sh1miR) and *gatgaagccactaccctatt* (sh2).

More details are available upon request.

Transient transfection

Transient transfection was performed using either Lipofectamine 2000 (Invitrogen) according to the manufacturer's instructions (scaling down one level), jetPEI (Polyplus-transfection) or Polyethylenimine (PEI; linear, MW ~25,000; Polysciences, Cat. #23966), as described in [9]. For co-transfection experiments, the plasmids were used at 1:1 ratio unless otherwise stated.

Lentivirus production and transduction

293T cells were transiently transfected with a lentiviral vector, the packaging and the envelope plasmids in the ratio of 2:1:1 using Lipofectamine 2000 or PEI. Scheme 1: 6–8 hours post-transfection, media were changed and cells were incubated at 32°C overnight for virus production; ~24 hours post-transfection, virus-containing media were filtrated through

0.45-µm PVDF filters (Carl Roth GmbH & Co. KG) with sterile syringes. Scheme 2: 6–8 hours post-transfection, cells were switched to 32°C and incubated overnight without medium change; ~24 hours post-transfection, cells were replenished with fresh media and incubated at 32°C for another 24 hours before virus harvest. Filtrated viruses were either used for transduction immediately or stored at -80°C for future use. We note that virus titer from scheme 2 was usually much higher than that from scheme 1.

For infection, culture media of target cells were replaced with virus-containing media and cells were incubated at 32°C or 37°C overnight. 48–72 hours post-transduction, cells were selected with 4 µg/ml puromycin (PAA Laboratories GmbH) or 10 µg/ml blasticidin (InvivoGen) for 3–7 days. Non-transduced cells were used as selection control. Of note, transduction at 37°C resulted in a better efficiency than at 32°C from our experience.

Preparation of cell extracts for Western blotting analysis

Cells were lysed on plates with either 2× SDS sample buffer (100 mM Tris-Cl, pH 6.8, 4% SDS, 0.01% bromophenol blue, 20% glycerol) supplied with 30–50 mM DTT, or with lysis buffers for pulldown or co-immunoprecipitation (Co-IP). If lysed with a lysis buffer, a portion of clarified lysates were further mixed with equal or higher volume of 2× SDS sample buffer supplied with 30–50 mM DTT. Samples were boiled at 98°C for 8–10 min before being loaded into an SDS-PAGE gel.

Western blotting/Immunoblotting

Following SDS-PAGE, proteins were transferred onto nitrocellulose membranes by tank blotting overnight. Membranes were blocked for 1 hour in blocking buffer (5% non-fat milk in TBS/0.1% Tween [TBS/T]) and incubated with primary antibodies with appropriate dilutions in blocking buffer for 1–2 hours at room temperature, or overnight at 4°C. After 3–4× wash with TBS/T, membranes were incubated with HRP-conjugated secondary antibodies (1:1000–2000) for 1–2 hours at room temperature and washed 4–5 times with TBS/T. ECL solution (Thermo Scientific Pierce), and ECL-Hyperfilm (GE Healthcare) or Fujifilm Super RX-N, were used to detect the signals.

For sequential probing using antibodies from different species, before the second probing, signals from the first round of Western blotting were inactivated by incubation with 30% H_2O_2 at 37°C for 30 min [11].

Ponceau S staining

Membranes were washed 3–5 times with H_2O, stained with Ponceau S solution (0.1% [w/v] Ponceau S, 5% [v/v] acetic acid in dH_2O) for ~1 min, and then destained with H_2O to remove background staining. For complete destaining, membranes were washed several times with TBS/T with continuous shaking.

Active Ras pulldown with cell lysates

The procedure was performed as per GST pulldown (see later), albeit with different lysis/wash buffers and GST-Raf1 RBD (either from Pierce Active Ras Pull-Down Kit or homemade) or GST-BRaf RBD (homemade) as the bait.

In Fig. 1a, b, S1c, and S8c, the lysis/wash buffer was 25 mM Tris·HCl, pH 7.2, 150 mM NaCl, 5 mM $MgCl_2$, 1% NP-40 or 1% Triton X-100, and 5% glycerol. In Fig. 6d, the lysis/wash buffer was PBS, 2 mM $MgCl_2$, and 0.7% CHAPS. 1× cOmplete or cOmplete ULTRA protease inhibitor cocktail (EDTA-free; Roche Diagnostics) was included in the lysing step; 2mM DTT was also included in the lysing step in Fig. 6d.

PDGF treatment prior to active Ras pulldown

MSC cells were seeded on poly-L-lysine-coated plates and cultured for ~30 hours; growth media were replaced with DMEM:F12-HAM (1:1) plus 1 μM forskolin, then cells were incubated overnight for growth factor starvation. For PDGF stimulation, media was replaced with the stimulation medium (the starvation medium plus 10 ng/ml PDGF-BB [Calbiochem]) equilibrated at room temperature, and cells incubated at 37°C for intended durations. Media were then removed and cells washed once with ice-cold PBS and lysed on ice; lysates were clarified by centrifugation at 15,000–20,000 g for 15 min at 4°C for active Ras pulldown. Non-stimulated cells were processed likewise, without prior medium change before wash with PBS.

Proximity biotinylation by BioID2

Constructs were transfected into 293 cells cultured on 6-well plates with PEI at the ratio of 4:1 to DNAs. In Fig. S3a, 0.8 µg of EFS-OLLAS-BioID2, or 0.6 µg of EFS-OLLAS-BioID2-KRas4B + 0.2 µg of EFS-OLLAS, was transfected per well. In Fig. 2d, 0.4 µg of EFS-OLLAS-BioID2, or 0.3 µg of EFS-OLLAS-BioID2-KRas4B + 0.1 µg of EFS-OLLAS, was transfected per well as 1× DNA amount; the total amounts of PEI/DNA complexes were scaled down for 0.5× and 0.25× DNA transfection. Approximately 24 hours later, media were replenished with biotin added; ~48 hours after transfection, cells were washed once or twice with room-temperature PBS and lysed on ice with the lysis buffer (PBS, 2 mM $MgCl_2$, 1% CHAPS, 2 mM DTT and 1× cOmplete ULTRA protease inhibitor cocktail). Lysates were clarified as described above for streptavidin (High Capacity Streptavidin Agarose beads; Pierce) pulldown. After binding at 4°C for 2–3 hours with rotation, beads were washed three times with the lysis buffer plus 0.2% SDS (omitting DTT and protease inhibitors). The beads were then boiled in 2× SDS sample buffer at 98°C for 10 min for SDS-PAGE and Western blotting. Western blotting with streptavidin-HRP was described in [12].

Co-immunoprecipitation

The basic buffer was 50 mM Tris pH 7.5, 100 mM NaCl, 2 mM $MgCl_2$, or PBS, 2 mM $MgCl_2$. For RasGAP Co-IP, 2% n-Octyl-β-D-glucopyranoside (NOG; Carl Roth, Germany) was supplemented; for overexpressed merlin and RasGAP Co-IP, 2% NOG plus 1% Triton X-100 were supplemented in the lysis/binding buffer, 1% Triton X-100 plus 0.1% Sodium deoxycholate (DOC; Carl Roth, Germany) were supplemented in the washing buffer. 1× cOmplete protease inhibitor cocktail was included for all lysing steps, but omitted for washing steps. Briefly, cells were washed once with ice-cold PBS and lysed on ice; lysates were cleared by centrifugation at 15,000–20,000 g for 15 min at 4°C; for each IP, the supernatant was incubated with an antibody immobilized on Gamma-Bind G sepharose (GE Healthcare) for 0.5–2 hours with rotation at 4°C; the sepharose was spun down by centrifugation at 2,000 g for 30 s at 4°C and washed three or four times with ice-cold lysis buffer. IP were eluted with 2× SDS sample buffer by boiling, resolved on SDS-PAGE and immunoblotted with appropriate antibodies.

Small-scale purification of GST-tagged proteins from *E. coli* for pulldown

All the constructs were cloned in pGEX-4T-1. In brief, plasmids were transformed into *E. coli* BL21 (DE3) pLysS or Rosetta 2 (DE3); bacteria were grown in 5 ml of LB medium with 100 µg/ml ampicillin with shaking at 37°C, overnight. A 1-ml sample of each overnight culture was inoculated into 50 or 100 ml of 2× YT medium with 100 µg/ml ampicillin, and grown with shaking at 37°C for 6–10 hours until the *E. coli* density was close to saturation. The shaker temperature was lowered to 16°C and 0.2 mM IPTG was added to induce protein expression overnight. All subsequent steps were carried out at 4°C or on ice. Bacteria were pelleted by centrifugation for 3 min at 12,000 g, then either frozen at -80°C for later use or processed directly for lysis. For lysis, pellets were resuspended in ice-cold lysis buffer (PBS/1% Triton X-100/protease inhibitor cocktail, 1 ml for 50-ml culture) and lysed by sonication (30% amplitude, 10 s impulse, 10 s break, 1 min total impulse; Digital sonifier S-450D, Branson). Cell debris was pelleted by centrifugation at 15,000 g for 15–30 min. Glutathione Sepharose 4B Fast Flow (GE Healthcare) was pre-washed with PBS/0.1% Triton X-100 in 1.5-ml Protein LoBind tubes (Eppendorf), resuspended in the same buffer and aliquoted into 1.5-ml Protein LoBind tubes (~120 µl of the original Sepharose for 50-ml culture); then the buffer was removed. Clarified *E. coli* supernatants were incubated with Glutathione Sepharose with rotation overnight. Afterwards, the Sepharose was washed four times with the lysis buffer (no protease inhibitor), and washed two times with PBS/0.01% DOC. After the final wash, 150 µl of PBS/0.01% DOC/0.02% NaN_3 was added and the samples stored at 4°C. The purity and yield of the proteins (on beads) were determined by Coomassie Blue staining after SDS-PAGE, with BSA standards as reference. The proteins (on beads) were diluted with the storage buffer to similar concentrations for pulldown; the amount of Sepharose was adjusted with unused Glutathione Sepharose.

In our later work, purified proteins (on beads) were stored in PBS/0.05% Triton X-100/50% glycerol/5mM DTT at -20°C.

Preparation of *E. coli* lysates for Western blotting or pulldown

SBP-merlin (isoforms and mutants) were expressed in *E. coli*; the preparation of lysates was same as described above for small-scale purification of GST-tagged proteins, except that the bacteria were resuspended in PBS/0.6% CHAPS/protease inhibitor cocktail before sonication. The clarified lysates were stored at -80°C for later use.

GST pulldown

GST-tagged proteins expressed in *E. coli* were immobilized on glutathione 4B sepharose (GE Healthcare). GST pulldown was performed in essentially the same way as Co-IP. For merlin:RasGAP interaction, 2% NOG plus 1% Triton X-100 were supplemented in the lysis/binding buffer; 1% Triton X-100 plus 0.1% DOC was supplemented in the wash buffer. For merlin:Ras interaction, 0.025–0.05% DDM was supplemented in the binding/wash buffer. For experiments using CHAPS, 0.6–0.7% CHAPS was supplemented in the lysis/binding buffer.

Bimolecular fluorescence complementation analysis

One day before transfection, 2 × 10^5 293T cells were seeded onto poly-L-lysine-coated coverslips in 6-well plates. Cells were co-transfected with jetPEI and the bimolecular fluorescence complementation (BiFC) plasmids and pEYFP-C1 (as transfection control, 1/10 of the total transfected DNA). 48 hours after transfection, cells were washed with PBS and fixed with 4% paraformaldehyde for 10 min at room temperature. Cell membranes (outside) were stained with Wheat Germ Agglutinin-Texas Red-X Conjugate (Invitrogen); coverslips were then mounted onto slides with ProLong Gold Antifade Mountant (Invitrogen). After drying, slides were either subjected to fluorescence microscopy (Zeiss Axio Observer Z1, with ZEN2 software, Carl Zeiss) or stored at -20°C for later use. A duplicate of the transfections was used to verify protein expression by immunoblotting.

Large scale purification of GST-tagged proteins from *E. coli*

pGEX-4T-1-GAP (full-length RasGAP), pGEX-4T-1-GAP 456–1047, pGEX-4T-1-GAP 714–1047 and pGEX-4T-1-NF2 1–313 were used to purify the respective proteins employing the GST tag. The procedure was similar to that described above for small-scale purification, with the following modifications: pre-cultures were inoculated in 25 ml of LB medium with

ampicillin for overnight culture; the 25-ml pre-cultures were inoculated into 1 l of 2× YT medium without antibiotics and grown for 6–8 hours at 37°C to reach the desired *E. coli* density; induction was performed at 16°C overnight with 0.1 mM IPTG; bacteria were pelleted by centrifugation at 6,000 g (Sorvall RC-3B Plus centrifuge, DuPont) for 20 min; 10 ml of ice-cold lysis buffer was used to suspend bacteria prior to sonication (30% amplitude, 15 s impulse, 15 s break, 3 min total impulse); 1 ml of the original Sepharose for 1 l culture; lysate/Sepharose incubation was conducted in 15-ml tubes; Sepharose was washed three times with the lysis buffer and four times with PBS (no detergent); the GST tag was cleaved on beads by thrombin (Merck KGaA) at room temperature overnight, with rotation; the supernatants were transferred to new tubes and Benzamidine Sepharose (GE Healthcare Life Sciences) was used to remove thrombin; Benzamidine Sepharose was pelleted and the supernatants were transferred to new tubes; the final supernatants were concentrated with Amicon Ultra-4 Centrifugal Filters (Millipore). Protein yield and purity were examined by Coomassie Blue staining after SDS-PAGE and compared with BSA standards. Proteins were stored at -20°C in 50% glycerol and 1 mM DTT.

Large scale purification of His-tagged proteins from *E. coli*

pET15TEV-HRas 1–166 was used to express N-terminal His-tagged HRas 1–166 protein; pET30a-NF2 was used to express N-terminal His-S-tagged full-length merlin. The procedure was similar to large scale purification of GST-tagged proteins, with the following modifications: for merlin purification, the lysis buffer was PBS with 1% Triton X-100, 1 mM DTT, 30 mM imidazole and the protease inhibitor cocktail; for HRas 1–166 purification, the lysis buffer was further supplemented with 2.5 mM $MgCl_2$ and 100 µM GDP; protease inhibitor and GDP were omitted for wash; Ni-Sepharose 6 Fast Flow (GE Healthcare) was used for binding His-tagged proteins; for HRas 1–166, ProTEV Protease (Promega) was used to cleave the His-tag on beads (ProTEV Protease has a His tag and so is retained by Ni-Sepharose); for merlin, it was either eluted with PBS/500 mM imidazole and dialyzed to remove imidazole, or the His tag was cleaved on beads with thrombin as described for

cleaving GST tag; for wash and storage of HRas 1–166, 2.5 or 2 mM $MgCl_2$ was always included.

Purification of SBP-tagged proteins from *E. coli*

The procedure was similar to that described above for small-scale purification, with the following modifications: Streptavidin Agarose was used to bind SBP-tagged protein; the proteins were eluted with biotin elution buffer (25 mM Tris pH 7.5, 150 mM NaCl, 5 mM biotin, 2 mM DTT, 50% glycerol) at 4°C or -20°C, and the supernatants containing eluted proteins were transferred to new Protein LoBind tubes and stored at -20°C.

Nucleotide loading into purified HRas 1–166

In brief, 200 µM HRas 1–166 was incubated with 2 mM GTPγS (non-hydrolyzable analog of GTP) or GDP in 100 µl of nucleotide loading buffer (20 mM Tris-HCl pH 7.5, 50 mM NaCl, 2 mM EDTA, 1 mM DTT) for 15 min at 30°C. Nucleotide loading was stopped by adding 1 M $MgCl_2$ to final 10 mM, with a 5-min incubation at room temperature. Nucleotide-loaded Ras protein was aliquoted and stored at -20°C.

Far-western blotting

GST-RasGAP fragments were separated by SDS-PAGE and transferred onto a nitrocellulose membrane. The membrane was stored in TBS/T at 4°C for one day to allow certain spontaneous refolding of the proteins. The membrane was blocked with 5% milk in TBS/T for 1 hour, and incubated with purified His-S-merlin (2 µg/ml in PBS/0.01% DOC) for 1 hour at room temperature. Afterwards, the membrane was washed 3 times with PBS/0.01% DOC. Bound merlin was detected with NF2 (B-12) antibody, like a routine immunoblotting. The GST-fusion protein inputs were detected by Ponceau S staining.

***In vitro* Ras GAP activity assay by GST-Raf1 RBD pulldown**

All proteins were purified from *E. coli*. Equal molar amount of RasGAP fragments (1 µg for FL RasGAP) were pre-incubated with or without 1 µg of merlin in 10 µl of GAP reaction buffer (50 mM Tris pH 7.5, 148 mM KCl, 2.5 mM $MgCl_2$, 1 mM DTT), containing 2 µg of BSA in 1.5 ml-protein LoBind tubes (Eppendorf), for 1 hour at room temperature and transferred on ice; 1.6 µg of HRas 1–166 was preloaded with 1 mM GTP in 20 µl of buffer (50 mM Tris

pH 7.5, 148 mM KCl, 1 mM DTT, 1 mM EDTA) for 15 min at 30°C, then 1 µl of 0.2 M $MgCl_2$ was added to stop the GTP loading reaction; GTP-loaded Ras was diluted with ice-cold 300 µl of GAP reaction buffer; 20 µl of diluted Ras-GTP (containing 0.1 µg Ras) was aliquoted into the reaction tubes on ice. The reaction was initialized by placing the tubes in a 37°C water bath and stopped by placing them back on ice. 1 ml of ice-cold pulldown buffer (50 mM Tris pH 7.5, 150 mM NaCl, 2 mM $MgCl_2$, 0.05% DDM) was immediately added into each reaction tube, then GST-Raf1 RBD immobilized on Sepharose was added. The samples were incubated for 1–2 hours with rotation at 4°C and continued as routine GST pulldown.

PBS/5 mM EDTA treatment for signaling analysis

Cells were plated onto 10-cm dishes to reach ~80% confluence. Culture medium was removed and cells were washed once with pre-warmed PBS. 5 ml of pre-warmed PBS/5 mM EDTA was added and cells then incubated at 37°C. At planned time points, the dishes were rapidly placed on ice and 10 ml of ice-cold PBS (for each dish) was added. Cells were immediately harvested with a cell scraper and transferred into pre-chilled 50-ml tubes. For control treatment (time point “0”), after wash with PBS, dishes were replenished with pre-warmed PBS instead of PBS/EDTA and immediately placed on ice; ice-cold PBS was further added and cells were harvested as described above. Cells were centrifuged at 4500 g, 4°C for 5 min; the pellets were dissolved in SDS lysis buffer (2% SDS, 62.5 mM Tris-HCl pH 6.8, 10% glycerol) supplemented with cOmplete proteinase inhibitor cocktail (EDTA-free) and PhosSTOP (Roche Diagnostics), and subjected to Western blotting.

Supplementary References

1 Widmann C, Gibson S, Johnson GL. Caspase-dependent cleavage of signaling proteins during apoptosis. A turn-off mechanism for anti-apoptotic signals. *J Biol Chem* 1998; 273: 7141-7147.

2 Orthaus S, Klement K, Happel N, Hoischen C, Diekmann S. Linker histone H1 is present in centromeric chromatin of living human cells next to inner kinetochore proteins. *Nucleic Acids Res* 2009; 37: 3391-3406.

3 Geissler KJ, Jung MJ, Riecken LB, Sperka T, Cui Y, Schacke S *et al*. Regulation of Son of sevenless by the membrane-actin linker protein ezrin. *Proc Natl Acad Sci U S A* (Research Support, Non-U.S. Gov't) 2013; 110: 20587-20592.

4 Riecken LB, Tawamie H, Dornblut C, Buchert R, Ismayel A, Schulz A *et al*. Inhibition of RAS activation due to a homozygous ezrin variant in patients with profound intellectual disability. *Hum Mutat* 2015; 36: 270-278.

5 Smith MJ, Ikura M. Integrated RAS signaling defined by parallel NMR detection of effectors and regulators. *Nature chemical biology* 2014; 10: 223-230.

6 Johannessen CM, Boehm JS, Kim SY, Thomas SR, Wardwell L, Johnson LA *et al*. COT drives resistance to RAF inhibition through MAP kinase pathway reactivation. *Nature* 2010; 468: 968-972.

7 Zufferey R, Nagy D, Mandel RJ, Naldini L, Trono D. Multiply attenuated lentiviral vector achieves efficient gene delivery in vivo. *Nat Biotechnol* 1997; 15: 871-875.

8 Stewart SA, Dykxhoorn DM, Palliser D, Mizuno H, Yu EY, An DS *et al*. Lentivirus-delivered stable gene silencing by RNAi in primary cells. *RNA* 2003; 9: 493-501.

9 Cui Y, Morrison H. Construction of cloning-friendly minigenes for mammalian expression of full-length human NF1 isoforms. *Hum Mutat* 2019; 40: 187-192.

10 Kim DI, Jensen SC, Noble KA, Kc B, Roux KH, Motamedchaboki K *et al*. An improved smaller biotin ligase for BioID proximity labeling. *Mol Biol Cell* 2016; 27: 1188-1196.

11 Sennepin AD, Charpentier S, Normand T, Sarre C, Legrand A, Mollet LM. Multiple reprobing of Western blots after inactivation of peroxidase activity by its substrate, hydrogen peroxide. *Anal Biochem* 2009; 393: 129-131.

12 Cui Y, Ma L. Sequential use of milk and bovine serum albumin for streptavidin-probed western blot. *Biotechniques* 2018; 65: 125-126.

13 Koonin EV, Mushegian AR, Tatusov RL, Altschul SF, Bryant SH, Bork P *et al*. Eukaryotic translation elongation factor 1 gamma contains a glutathione transferase domain--study of a diverse, ancient protein superfamily using motif search and structural modeling. *Protein Sci* 1994; 3: 2045-2054.
